# Proteotoxic stress promotes entrapment of ribosomes and misfolded proteins in a shared cytosolic compartment

**DOI:** 10.1093/nar/gkaa068

**Published:** 2020-02-07

**Authors:** Arnab Ghosh, Loren Dean Williams, Dimitri G Pestov, Natalia Shcherbik

**Affiliations:** 1 Department for Cell Biology and Neuroscience, Rowan University, School of Osteopathic Medicine, 2 Medical Center Drive, Stratford, NJ 08084, USA; 2 School of Chemistry and Biochemistry, Georgia Institute of Technology, 315 Ferst Drive NW, Atlanta, GA 30332, USA

## Abstract

Cells continuously monitor protein synthesis to prevent accumulation of aberrant polypeptides. Insufficient capacity of cellular degradative systems, chaperone shortage or high levels of mistranslation by ribosomes can result in proteotoxic stress and endanger proteostasis. One of the least explored reasons for mistranslation is the incorrect functioning of the ribosome itself. To understand how cells deal with ribosome malfunction, we introduced mutations in the Expansion Segment 7 (ES7L) of 25S rRNA that allowed the formation of mature, translationally active ribosomes but induced proteotoxic stress and compromised cell viability. The ES7L-mutated ribosomes escaped nonfunctional rRNA Decay (NRD) and remained stable. Remarkably, ES7L-mutated ribosomes showed increased segregation into cytoplasmic foci containing soluble misfolded proteins. This ribosome entrapment pathway, termed TRAP (Translational Relocalization with Aberrant Polypeptides), was generalizable beyond the ES7L mutation, as wild-type ribosomes also showed increased relocalization into the same compartments in cells exposed to proteotoxic stressors. We propose that during TRAP, assembled ribosomes associated with misfolded nascent chains move into cytoplasmic compartments enriched in factors that facilitate protein quality control. In addition, TRAP may help to keep translation at its peak efficiency by preventing malfunctioning ribosomes from active duty in translation.

## INTRODUCTION

An essential role of the ribosome is to carry out protein synthesis. Besides the translation of genetic information, the eukaryotic ribosome also provides a platform for the initial folding of nascent polypeptide chains, their post-translational modifications and correct intracellular targeting (reviewed in (1)). To maintain proteostasis and avoid aberrant-rRNA-directed accumulation of misfolded, improperly modified and mistargeted polypeptide chains, cells have evolved a diverse repertoire of co- and post-translational protein quality control (PQC) mechanisms, which eliminate anomalous polypeptides with the help of molecular chaperones, enzymes of the ubiquitin-proteasome system (UPS) and autophagy (2). Several recent studies have demonstrated that anomalous polypeptides that escape PQC are sequestered into cytoplasmic protein inclusions such as iPOD, INQ, Q bodies, JUNQ, VHL puncta, stress bodies, peripheral aggregates and CytoQ (3–10). It is thought that sequestration of anomalous polypeptides in subcellular compartments is an important cellular strategy that supplements PQC (11).

Mistranslation might be caused in some cases by defects of the ribosome itself. Given the staggering complexity of this molecular machine (12,13), a diversity of mutations or molecular lesions of the ribosome could lead to synthesis of anomalous proteins, endangering cell proteostasis. Indeed, previous studies have uncovered surveillance mechanisms that monitor ribosome biogenesis and prevent the release of faulty ribosomes into the translating pool (14–16). In contrast, much less is known about the molecular mechanisms that underlie the surveillance for malfunctional ribosomes that are assembled and actively involved in translation. One late-acting ribosome surveillance mechanism described in prokaryotes was shown to operate on assembled 70S ribosomes composed of a non-functional rRNA-mutated subunit paired with a wild-type subunit (17,18). In eukaryotes, nonfunctional ribosome subunits were shown to be selectively targeted for decay (19,20). To date, studies of the nonfunctional rRNA decay (NRD) pathway in the *Saccharomyces cerevisiae* have focused on mutations in the peptidyl-transferase center (PTC) of the 60S or the decoding site (DCS) of the 40S subunit, which trigger two distinct decay processes (18S-NRD and 25S-NRD, respectively) taking place at different subcellular locations (19,20). The 40S subunits with nonfunctional DCSs were observed to localize to processing bodies (P-bodies), wherein their rRNA is degraded by Xrn1 and the exosome complex (19). The 40S ribosomal protein uS3 (Rps3) plays an important role in 18S-NRD (21), as it undergoes sequential ubiquitination in stalled ribosomes, followed by 40S release from 60S subunits and Xrn1-assisted degradation of 18S rRNA (22). The 60S subunits carrying mutations in the PTC were reported to undergo polyubiquitination and Cdc48-mediated dissociation from 40S subunits (23). These nonfunctional 60S subunits were found in the perinuclear region in yeast cells (19), presumably as part of a degradation process that involves the proteasome (23).

Despite progress in studies of the NRD triggered by defects in the PTC and DCS, many important questions remain. Can NRD target any type of functionally impaired ribosomal subunits? How efficiently does this pathway recognize and eliminate ribosomes with mutations that affect the ribosome's auxiliary functions, such as interactions with the post-translational protein-folding machinery? Do PQC and ribosome surveillance mechanisms communicate and/or collaborate? We reasoned that one approach to start addressing these questions would be to study mutations in the rRNA Expansion Segments (ESs). ESs represent blocks of rRNA gained by eukaryotes during evolution (24) that are located on the solvent-exposed surfaces of the ribosome, away from the core functional centers such as the PTC and DCS (25). Although the function of many ESs remains unknown, a deletion analysis of ESs in yeast ribosomes revealed that many of them are essential for viability, pointing to important roles in the cell (26). Among nonessential ESs, ES27L has been recently shown to provide a platform for key enzymes responsible for nascent peptide modification and processing, thereby contributing to translational fidelity (27,28).

In this study, we focused on expansion segment 7 (ES7L) in the eukaryotic ribosome, prompted in part by our recent work showing links between ES7L, oxidative stress adaptation, and cell survival in yeast (29,30). Previous structural evidence (31–33) suggested that ES7L functions as a regulatory region in the ribosome. Consistent with large-scale proteomics studies (34) and mRNA interactome analyses (35), biochemical pulldowns identified a number of proteins, including aminoacyl-tRNA-synthetases, chaperones and metabolic proteins, that potentially interact with 60S subunits via ES7L (36), further implying that ES7L is involved in regulatory functions of the ribosome. Here, we identified rRNA mutations in the conserved central junction of ES7L (ES7L_CJ_) that allow ribosomes to participate in translation but render them functionally defective and unable to support cell viability. Although malfunctional, these mutant 60S subunits escape NRD and remain stable in cells. Remarkably, mutant subunits segregate into cytoplasmic areas enriched in soluble misfolded proteins. These results shed new light on the interplay between protein and ribosome quality control in maintaining cellular proteostasis. We discuss how Translational Relocalization with Aberrant Polypeptides (TRAP) may serve the dual role of eliminating aberrant nascent polypeptides and restricting malfunctioning ribosomes to mitigate their detrimental impact on translation.

## MATERIALS AND METHODS

### Yeast strains and culture manipulations

Wild-type BY4741 (*MAT*a *his3-1 leu2-0 met15-0 ura3-0*) and its derivative deletion strains (*ltn1*Δ and *vms1*Δ) were obtained from Thermo Fisher. The NOY891 strain (*MAT***a***ade2-1 ura3-1 trp1-1 leu2-3,112 his3-11 can1-100 rdnΔΔ::HIS3*) carrying pNOY353 was kindly provided by Masayasu Nomura. We used standard recipes for YPDA (1% yeast extract, 2% peptone, 2% dextrose, 10 mg/l adenine), YPGal (1% yeast extract, 2% peptone, 2% galactose, 1% raffinose, 10 mg/l adenine) and synthetic dropout media. Yeast transformations were done using a standard protocol (37). BY4741 cells transformed with the pDP333 plasmid and its derivatives were plated on dextrose synthetic dropout plates supplemented with doxycycline (Dox), while NOY891 transformants were plated on galactose synthetic dropout Dox-supplemented plates. To monitor colony variability and avoid occasional colonies that have abnormal deviations in the plasmid-driven rRNA expression levels, we routinely re-streaked at least four individual colonies from each transformation on appropriate agar plates supplemented with Dox and verified the expression of 18S^tag^ and 25S^tag^ rRNAs. To do so, individual colonies picked from each streak were grown to mid-log phase in liquid SD-leu^−^ without Dox at 30°C. Total RNA was then extracted and analyzed by northern hybridizations with probes FL127 and FL128 (Supplementary Table S1).

All manipulations with yeast cells and cultures were done identically between the samples compared in each experiment. Unless indicated otherwise, yeast cultures were grown from a single colony in synthetic medium lacking appropriate amino acids overnight (∼16 h) at 30°C, diluted with fresh medium to OD_600_ ∼0.2 and grown for an additional 3 h at 30°C before each experiment. For the transcriptional shut-off experiments, cells were grown overnight, diluted to OD_600_ ∼0.2, grown for 3 h, and Dox was added to the cultures. Aliquots of cells were taken every 30 min for the next 2 h and RNA was immediately extracted by the FAE method (38). For P-body detection, BY4741 cells harboring pDP333 constructs were co-transformed with pRS313-DCP2-GFP, in which P-body marker Dcp2-GFP is constitutively expressed from the endogenous promoter. Transformants were plated on SD-leu^−^his^−^ agar plates supplemented with Dox, a single colony was grown overnight in SD-leu^−^his^−^ medium without Dox at 30°C, diluted to OD_600_ ∼0.2 and grown for an additional 3 h. To reduce stress caused by medium change, cells were washed within ∼1 min and quickly resuspended in pre-warmed SD-leu^−^his^−^ or in minimal medium lacking dextrose and grown for 30 min at 30°C. For iPOD and VHL puncta detection, BY4741 cells harboring pDP333 constructs containing wild-type or A501U 35S pre-rRNA were co-transformed with pYes- Htt103QP-GFP or PESC-URA-VHL-GFP plasmids, in which the Htt103QP-GFP and the VHL-GFP are cloned under the GAL1 promoter. Transformants were plated on SD-leu^−^ura^−^ agar plates supplemented with Dox, a single colony from each plate was then grown in SD-leu^−^ura^−^ overnight at 30°C, diluted to OD_600_ ∼0.2, quickly washed, resuspended in pre-warmed galactose-containing minimal medium and grown for an additional 5 h. To induce heat shock in VHL- GFP-expressing cells, one half of the culture was shifted to 37°C for 30 min.

### Cell viability and growth assays

For cell viability assays, NOY891 strains carrying pNOY353 and pDP333 plasmids were grown overnight at 30°C in galactose-containing minimal medium lacking leucine and tryptophan and supplemented with Dox. Cultures were diluted with the same media to OD_600_ ∼0.2 and grown for an additional 4 h at 30°C. Cells were divided into three subcultures, washed, adjusted to the same concentration of 2 × 10^6^ cells/ml and six 1:5 serial dilutions were plated on YPDA, YPDA + Dox or YPGal agar plates. Plates were incubated at 30°C for 5–7 days before their images were taken.

For growth assays, yeast cultures were adjusted to an OD_600_ ∼0.2 with SD-leu^−^ media, 200 μl of each culture were inoculated into 96-well plates in three replicates. The cultures were grown for 30 h at 30°C with shaking. OD_600_ measurements were taken every 5 min and automatically recorded using a BioTek Synergy HT microplate reader. The doubling time was defined as described in (39).

### Plasmids

To generate constructs for Dox-regulated expression of tagged rRNAs, pJD694 (a kind gift from Jonathan Dinman (40)), was cut with BglII and the excised portion of rRNA replaced with a 4.5 kb BglII fragment of pWL160 (a kind gift of Melissa Moore (20)) containing tags within the rRNA coding sequence. The resulting construct pDP315 was linearized by partial digestion with EcoRV, which cleaves within the *URA3* marker in this plasmid, the full-sized DNA was purified from agarose gel and cotransformed into BY4741 cells together with a PCR-generated *LEU2* sequence flanked by regions identical with the pDP315 sequence to replace *URA3* with *LEU2* by homologous recombination, thus generating the final construct pDP333. We used NEBuilder^®^ HiFi DNA Assembly Cloning (New England Biolabs) for the incorporation of point nucleotide substitutions in the pDP333 plasmid. All other plasmids used in this study are listed in the Supplementary Table S2.

### Chemicals and reagents

Cycloheximide (CHX) was purchased from Sigma and used at concentrations 50 or 100 μg/ml; doxycycline was purchased from Frontier Scientific and used at 10 μg/ml. Mounting solution (ProLong™ Gold Antifade Mountant) was from Thermo Fisher Scientific. Mouse monoclonal anti-Nop1 antibodies (MCA-28F2) from EnCor Biotechnology were diluted 1:250 prior to use; monoclonal anti-GFP antibodies were from Santa Cruz Biotechnology (sc-9996) and used at 800 ng/ml, Alexa 488-conjugated secondary antibodies were purchased from Invitrogen and used at 1:1000 dilution.

### Structural modeling and RNA folding analyses

Structure of the yeast 80S ribosome (PDB file 4V88) (33), *Drosophila melanogaster* ribosome (PDB file: 4V6W) (41) and human 80S (PDB file: 6QZP) (42) were obtained from the Protein Data Bank and visualized with PyMOL 2.1 (Schrödinger, https://pymol.org/2/). Putative RNA secondary structures of the mutant ES7L regions were obtained using Mfold (http://unafold.rna.albany.edu/?q=Mfold/RNA-Folding-Form) and analyzed as described in (43).

### RNA extraction, northern blotting, signal quantification and RT-qRCR

Total RNA extraction was done by one-step FAE technique as described previously (38). We follow the procedure described in (44) for RNA separation and the procedure described in (45) for Northern hybridizations. Sequences of all probes and primers used in this study are listed in Supplementary Table S1. Signal quantification was done as described in (29). For more details, please refer to Supplementary Data section.

RT-qPCR was performed as described in (29). The detailed description of RT-qPCR following MIQE guidelines (46) can be found in the Supplementary Data section. Primer's sequences are listed in Supplementary Table S3. All samples were analyzed in triplicate, and *ACT1* was used to normalize the mRNA expression levels.

RT-qPCR-based technique used for quantification of the relative content of the endogenous and tagged rRNA can be also found in Supplementary Data section.

### Sucrose gradient centrifugation analysis

Sucrose gradient centrifugation analysis was performed as described in (47). The detailed description of this technique can be found in the Supplementary Data section.

### Fluorescence *in situ* hybridizations, immunostaining, microscope image acquisition and analysis

Fluorescent in situ hybridization was done as described in (48) with a few minor modifications. Cells were grown at 30°C overnight, diluted to OD_600_ ∼0.2 in the appropriate medium, grown for an additional 3 h and harvested by centrifugation at 3000 × g for 3 min. Drug treatment conditions are indicated in figure legends. Cells were fixed for 1 h at 30°C in a 4% formaldehyde solution prepared in phosphate buffer (100 mM potassium phosphate, pH 6.5). Cells were washed, resuspended in 1.2 M sorbitol prepared in the phosphate buffer, and treated with Zymolyase (Sunrise Science Products) for 30 min at room temperature. Spheroplasts were plated on poly-lysine coated glass slides and pre-hybridized at 37°C for 1 h in the hybridization buffer containing 0.02% Denhardt's solution, 4× SSC, 50% formamide, 10% Dextran sulfate, 0.75 mg/ml salmon sperm DNA, and 0.4 U/μl Ribolock (Thermo Fisher). Fluorescent probes were added to the hybridization buffer at the concentration of 0.2 μM and incubated with cells in a humid dark chamber at 37°C overnight. Cells were then washed with 2× SSC for 30 min, 1× SSC for 20 min and 0.5× SSC for 20 min. Immunostaining was done as previously described (49). In brief, the FISH hybridized cells were washed once with PBS containing 0.2% Tween 20 (PBST), blocked for 30 min with 2% BSA fraction V (Sigma) prepared in PBST, hybridized with primary antibodies for 1 h at room temperature, washed and incubated with fluorophore-conjugated secondary antibodies for 1 h at room temperature. All antibodies used in this analysis were diluted 1:250 in PBST/BSA buffer. Prior to the addition of the mounting solution, cells were stained with DAPI to visualize nuclei. Microscopic images were acquired with an Axiovert inverted microscope (Carl Zeiss Microscopy, LLC) using a 63× objective. Images were analyzed using Zen Blue software (Zeiss).

## RESULTS

### Design of ES7L mutants and characterization of their expression in cells

**Figure 1.**
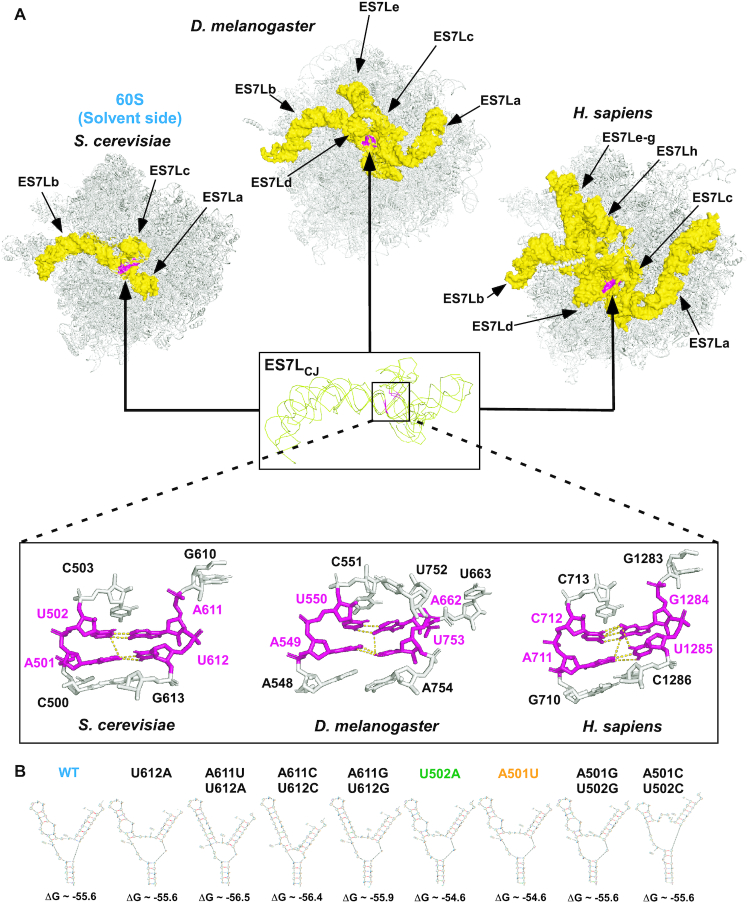
Yeast ES7L contains a region evolutionary conserved in eukaryotes. (**A**) View of ES7L from the 60S solvent side in budding yeast (*Saccharomyces cerevisiae*), fruit fly (*Drosophila melanogaster*) and human (*Homo sapiens*) depicting the progressively increasing complexity of ES7L. Different tentacle-like rRNA extensions (yellow) protrude from the invariant central junction (ES7L_CJ_, shown enlarged as superimposition of structures from the three species). The highly conserved part of ES7L_CJ_ corresponding to nucleotide pairs A501:U612 and U502:A611 of budding yeast is shown in magenta, surrounding bases are shown in more detail at the bottom. Dashed lines indicate predicted polar contacts between the four conserved bases (A501:U612 and U502:A611). The following PDB files were used: *S. cerevisiae*, 4V88; *D. melanogaster*, 4V6W; *H. sapiens*, 6QZP. (**B**) Secondary structures of the wild-type and mutant ES7L as predicted by Mfold.

ES7L is located on the surface of the large (60S) ribosomal subunit, bordering the central protuberance (33). ES7L is an extension of Helix 25 of the prokaryotic ribosome that expanded during evolution to become one of the largest, most structurally complex, and diverse ribosomal segments in eukaryotes (Figure 1A and (26,36)). Most of this variability stems from the branched rRNA extensions that in metazoans acquire tentacles (24), protruding from the conserved central junction (ES7L_CJ_). As shown in Figure 1A, ES7L in fruit flies and humans is made up of five (a–e, total ∼348 nt long) and eight (a–h, total ∼866 nt long) extensions, respectively, as compared to only three (a–c, total ∼210 nt long) extensions seen in yeast. Despite this variability, the structure of the central junction region of ES7L corresponding to nucleotide pairs A501:U612 and U502:A611 (yeast numbering) is highly conserved between eukaryotes (Figure 1A). We sought to determine the effects of mutations in this part of yeast ES7L, as the deletion of the entire ES7L led to a lethal phenotype due to ribosome biogenesis defects and pre-rRNA degradation (26). We designed a set of mutants containing one or two nucleotide substitutions within the nucleotide pairs A501:U612 and U502:A611 (Figure 1B) and examined them using Mfold. Eight ES7L_CJ_ mutants with a thermodynamic profile similar to that of the unaltered ES7L_CJ_ (ΔGs, Figure 1B) were selected for further investigation. In the Mfold models, the secondary structures of the mutants showed minor changes in the size and shape of the junction loop and/or length of the ES7Lc tentacle when compared to wild-type ES7L (Figure 1B).

**Figure 2.**
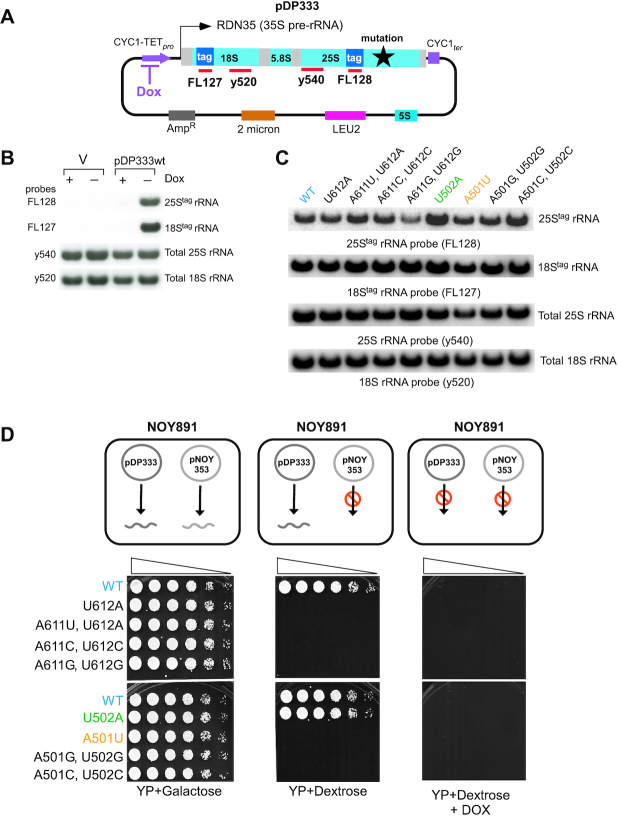
Design, validation of expression, and viability of mutant tagged rRNAs. (**A**) Schematic representation of the pDP333 plasmid. Doxycycline (Dox)-repressible promoter (CYC1-TET*_pro_*) and CYC1 terminator (CYC1*_ter_*) flank the 35S pre-rRNA coding sequence *RDN35*. Probes FL127 and FL128 allow specific detection of plasmid-derived rRNAs through short tag sequences inserted into the 18S and 25S rRNA coding sequences, respectively. Probes y520 and y540 detect both endogenous and plasmid-derived rRNA. pDP333 also carries a copy of the *RDN5* gene for 5S rRNA, a *LEU2* selectable marker, 2μ DNA replication origin and ampicillin resistance gene. (**B**) BY4741 cells transformed with pNS1 (empty vector control, V) or pDP333 containing tagged wild-type rRNA were grown in SD leucine dropout (SD-leu-) media with (+) or without (−) Dox, total RNA was extracted and analyzed by northern hybridizations with probes indicated on the left. (**C**) BY4741 cells transformed with pDP333 plasmids containing the indicated mutations were grown as in (B) without Dox, RNA was analyzed by northern hybridizations. (**D**) *Top panel*: Using media of different composition with the NOY891 strain allows two different plasmids, pDP333 and pNOY353, to be used as the source of rRNA. *Bottom panel*: NOY891 strain carrying pNOY353 to express wild-type rRNAs from a GAL1 promoter was transformed with pDP333 plasmids carrying mutations indicated on the left. Transformants were grown in SD-leu-ura- in the presence of Dox at 30°C, adjusted to the same cell density and their five-fold dilutions were plated on agar media indicated at the bottom. Plates were incubated at 30°C for 5–7 days.

To express rRNAs of interest (wild-type or mutant) in *S. cerevisiae*, we adopted and modified a previously published construct (40). We expressed 35S precursor rRNA (pre-rRNA) from a 2μ *LEU2* plasmid under the control of a doxycycline (Dox)-repressible Pol II promoter (pDP333, Figure 2A). In this construct, expression of the 35S pre-rRNA can be turned on and off by removal and addition of Dox, respectively (40). This approach allows for tight control of expression of plasmid-borne RNAs and minimizes the frequency of secondary mutations in these RNAs (40). To distinguish between endogenous and plasmid-borne rRNAs, two short tag sequences were inserted into the 18S and 25S portions of 35S pre-rRNA (Figure 2A) (20). To validate this system, the wild-type yeast strain BY4741 was transformed with the 2μ *LEU2* plasmid pNS1, used in this experiment as an empty vector control, or with pDP333, and grown overnight in the presence or absence of Dox. Total cellular RNA was analyzed by Northern hybridization using probes against plasmid-derived tags placed on 25S (FL128) and 18S (FL127) rRNAs, as well as with probes against endogenous 25S (y540) and 18S (y520) rRNAs (Figure 2A). As expected, tag-specific probes detected the expression of 18S^tag^ and 25S^tag^ rRNAs when cells were grown in Dox-free medium but not in the presence of Dox. Rehybridization of the same blots with probes y540 and y520 confirmed comparable levels of endogenous 25S and 18S rRNAs in Dox-containing and Dox-free cultures (Figure 2B). By using RT-qPCR with primers specific for either endogenous (untagged) or plasmid-derived, tagged rRNAs, we estimated that in cells grown without Dox, 18S^tag^ rRNA accounted for ∼3%, and 25S^tag^ rRNA for ∼2% of the corresponding cellular rRNAs (Supplementary Figure S1).

Using the tagged-rRNA plasmid pDP333, we next generated a set of constructs containing single and double nucleotide substitutions within ES7L_CJ_ (Figure 1B). These plasmids were transformed into wild-type cells, and expression of the ES7L_CJ_ rRNA mutants was induced by switching cultures to Dox-free medium. Northern hybridization using 25S^tag^ rRNA probe FL128 revealed that 25S rRNAs carrying the mutation(s) in the ES7L_CJ_ region (Figure 1B) could be stably expressed. Notably, steady-state levels of different mutants showed some variability, implying altered rates of their synthesis and/or decay (Figure 2C).

These data suggest that point mutations in ES7L_CJ_ do not significantly affect pre-rRNA transcription, processing and 60S subunit biogenesis, and results presented below provide additional support for this conclusion.

### Phenotypic analysis of cells expressing ES7L-mutated 25S rRNAs

To determine whether ES7L-mutant ribosomes are functional, we first tested their ability to support cell growth. For this analysis, we transformed pDP333 constructs into the NOY891 strain that lacks endogenous rDNA and relies on the galactose-inducible expression of rRNAs from the pNOY353 plasmid (50). This system allowed us to turn off the expression of wild-type 25S rRNA from pNOY353 by shifting cells from galactose to dextrose in addition to regulating the expression of 25S^tag^ rRNA from pDP333 with Dox (Figure 2D). The NOY891 cells transformed with pDP333 constructs were grown in galactose medium containing Dox and then plated on different agar plates to express rRNA either from pDP333 or pNOY353 (as diagrammed in Figure 2D). As expected, suppression of rRNA expression from both pNOY353 and pDP333 with dextrose and Dox abolished cell growth (Figure 2D, right), whereas galactose medium, which allowed rRNA expression from both plasmids, supported growth (Figure 2D, left). In dextrose medium lacking Dox, pDP333 becomes the sole source of rRNA, revealing the impact of mutations on cell viability (Figure 2D, middle). Among the tested mutations, only one, U502A, was benign and permitted cell growth when compared with the wild-type 25S rRNA (Figure 2D, middle), while the other seven ES7L_CJ_ mutations were lethal (Figure 2D, middle). Thus, even subtle nucleotide substitutions within the ES7L signature fold can render ribosomes defective in function. However, none of these mutations conferred a detectable dominant-negative growth phenotype when coexpressed with wild-type pNOY353-derived rRNA on galactose-containing medium (Figure 2D, left) or when expressed in the wild-type BY4741 strain in a liquid culture (Supplementary Figure S3).

### The 25S^tag^-A501U rRNA is not a substrate of NRD

We focused on two ES7L mutants: viable U502A and nonviable A501U. Despite striking phenotypic differences between them (Figure 2D), neither mutation significantly perturbed the overall folding pattern of the central junction region of ES7L as predicted by Mfold (Figure 1B). In a previous study, single nucleotide substitutions in the PTC of 25S rRNA or the DCS in 18S rRNA were observed to promote the rapid degradation of the aberrant rRNAs through NRD (20). Therefore, we considered the possibility that ribosomes containing the nonviable A501U mutation might be degraded through NRD, whereas those with the benign U502A mutation are not NRD substrates.

**Figure 3.**
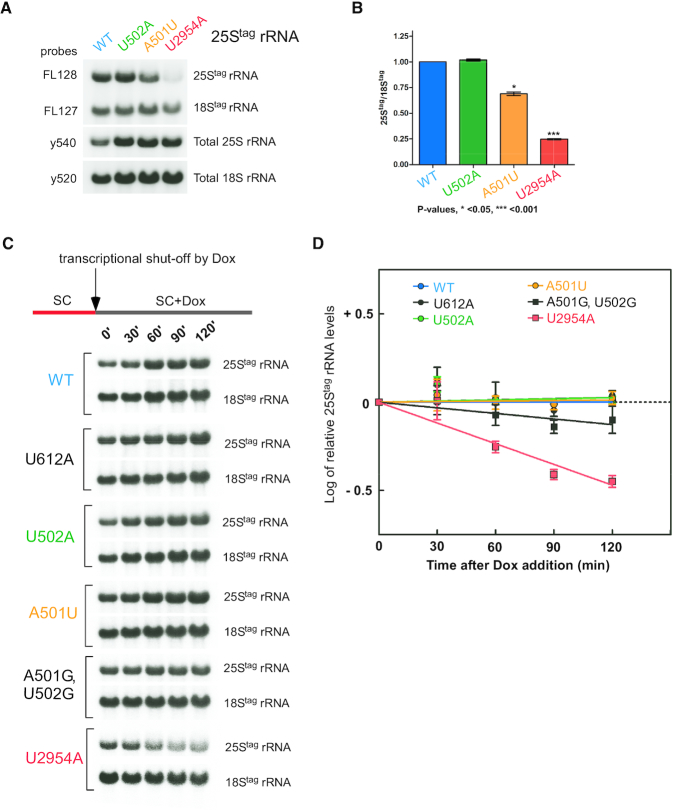
25S^tag^-A501U rRNA is more stable than the 25S-NRD substrate 25S^tag^-U2954 rRNA. (**A**) Overnight cultures of BY4741 transformed with pDP333 constructs carrying wild-type or one of the indicated mutant rRNAs were grown as in Figure 2C. rRNA was analyzed by Northern hybridizations with the indicated probes. (**B**) To quantify steady-state levels of the wild-type and mutant 25S^tag^ rRNAs, hybridization signals in (A) were converted to phosphorimaging units and used to calculate the 25S^tag^/18S^tag^ ratios. Mean values with standard errors were determined for three biological replicates and normalized to wild type. *P* values were determined by one-way ANOVA plus Dunnett's multiple comparisons test. (**C**) Expression of tagged rRNA was shut down at *t* = 0 with Dox in growing BY4741 cell cultures that carry 25S^tag^ wild-type and mutant rRNA constructs indicated on the left. RNA was extracted from equal volumes of cultures at each time point and analyzed by Northern hybridizations with probes FL127 and FL128 against tags placed in 18S and 25S rRNAs, respectively. Total rRNA levels in this experiment increase with time due to the continuing synthesis of endogenous rRNA in the cultures. (**D**) Stability of the mutant 25S^tag^-rRNAs after the Dox transcriptional shutdown. Levels of tagged mutant rRNAs were normalized to the average wild-type 25S^tag^ rRNA level at each time point and the log(2) of the difference plotted on the graph. Error bars show S.D. in three biological replicates. Quantification of the rRNAs was done by Northern hybridizations with probe FL128, one replicate of this analysis is presented in panel C.

Given the sensitivity of the yeast cellular rRNA levels to growth conditions (51) and variability of tagged rRNA expression between transformed yeast clones, one way to estimate rRNA stability is to quantify levels the of 25S^tag^ rRNA relative to the 18S^tag^ rRNA in samples isolated from the same cells. To this end, we shifted yeast transformants grown in the presence of Dox to Dox-free media for 16 h to induce tagged rRNA production. As in previous experiments (Figure 2B and C), the extracted RNA was hybridized with probes that detect total 25S and 18S rRNAs and plasmid-derived 25S^tag^ and 18S^tag^ rRNAs (Figure 3A). As a control, we used the canonical NRD substrate 25S^tag^-U2954A rRNA (previously referred to as U2585A using the *E. coli* nomenclature). In agreement with previous studies (20), the 25S^tag^-U2954A mutant exhibited a significant decrease in the 25S^tag^/18S^tag^ ratio (by ∼75%, Figure 3B). The viable U502A mutation did not reduce the 25S^tag^/18S^tag^ ratio relative to wild-type rRNA, while the 25S^tag^-A501U rRNA only showed a modest reduction (∼25%) (Figure 3A and B).

To more directly measure rRNA stability, we next performed a Dox shut-down experiment, in which we grew cells first in the absence of Dox to generate ribosomes containing tagged rRNAs, after which we added Dox to stop their new transcription. Hybridization analysis using wild-type plasmid-derived 25S^tag^ and 18S^tag^ rRNAs showed that the addition of Dox reduced accumulation of tagged rRNAs ∼10-fold (Supplementary Figure S2), whereas synthesis of endogenous rRNA continued unabated. To determine how ES7L mutations might affect rRNA stability, we shut down transcription of new plasmid-derived rRNAs by adding Dox to growing cultures that expressed 25S^tag^-A501U, 25S^tag^-U502A, 25S^tag^-U2954A and wild-type 25S^tag^ rRNAs. We harvested cells every 30 min and quantified rRNA amounts by hybridization with tag-specific probes (Figure 3C). This analysis showed that the level of the NRD substrate 25S^tag^-U2954A decreased in cells rapidly as expected, but the levels for single-nucleotide mutants U502A and A501U were indistinguishable from the wild-type 25S^tag^ rRNAs within this time frame (Figure 3D). Among other nonviable ES7_CJ_ mutants tested, the U612A and A501U mutations did not reduce stability relative to the wild-type 25S^tag^ rRNA. The double-nucleotide mutation A501G, U502G caused a moderate decline in stability that was less pronounced than that of the NRD mutation U2954A (Figure 3D). These results were corroborated by monitoring the 25S^tag^/18S^tag^ ratios (Supplementary Figure S4A) and the ratios of 25S^tag^ rRNA to U3 snoRNA in the same samples after Dox shut-down of tagged rRNA expression (Supplementary Figure S4B).

Based on this analysis, we conclude that ribosomes harboring the A501U mutation do not behave as a canonical NRD substrate and remain stable in cells over time. Thus, certain mutations in ES7L rRNA can render ribosomes uncapable of properly carrying out translation, based on their failure to support cell viability (Figure 2D), but do not mark these ribosomes for immediate degradation through NRD. Characterization of this new phenomenon was an objective of the investigation described in the following sections.

### Ribosomes harboring the A501U mutation in 25S rRNA are present among translating ribosomes

**Figure 4.**
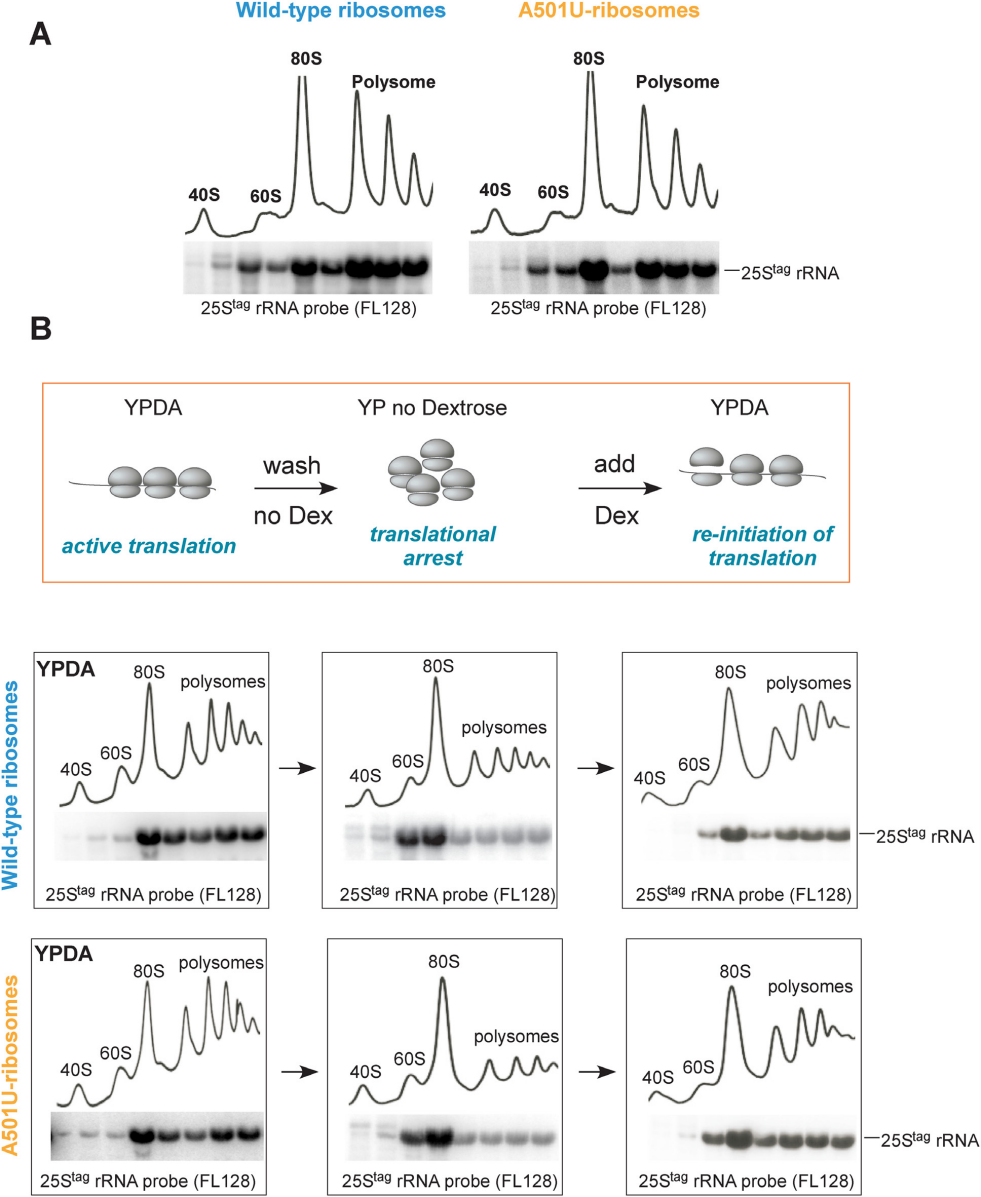
A501U-mutant ribosomes are translationally competent. (**A**) BY4741 cells transformed with pDP333 carrying wild-type 25S^tag^ rRNA or 25S^tag^-A501U rRNA were grown without Dox overnight, diluted in YPDA, grown for additional 2 h, harvested and lysed in buffer S (see Methods). Lysates were centrifuged through a 15–45% sucrose gradient. Gradients were fractionated with the continuous measurement of absorbance at 254 nm to visualize ribosomal peaks. Total RNA was extracted from individual fractions and analyzed by Northern hybridizations with the 25S^tag^ rRNA probe FL128. (**B**) Schematic workflow of the translational arrest and re-initiation assay. Cell cultures shown in (A) were grown without Dox overnight, diluted in YPDA, grown for an additional 2 h, and a portion of the culture was withdrawn for gradient analysis (left). Remaining culture was collected, washed twice, resuspended in YP medium lacking dextrose and grown for 30 min (middle). Dextrose was added back to the cultures; cells were grown for additional 30 min and harvested (right). Cells were lysed in buffer F (see Methods) and lysates were analyzed by sucrose gradients as in (A).

The first question addressed was whether ribosomes with the A501U mutation in 25S rRNA (hereafter, A501U-ribosomes) participate in translation. We resolved cell lysates prepared from exponentially growing cells expressing wild-type 25S^tag^ or 25S^tag^-A501U rRNAs by sucrose-gradient centrifugation and analyzed rRNA by Northern hybridizations. Both wild-type and A501U mutant rRNAs were present in the 60S, 80S and polysomal fractions (Figure 4A), indicating that the A501U nucleotide substitution within ES7L_CJ_ does not prevent 60S subunits from full maturation and translational competence. To confirm that A501U-ribosomes are true translating ribosomes, we increased the concentration of KCl in the lysis buffer to 400 mM to promote the dissociation of non-translating ribosomes not bound to mRNA (52) and analyzed the distribution of 25S^tag^-A501U rRNA across the sucrose gradient in comparison with wild-type 25S^tag^ rRNA. Consistent with a previous study (53), we observed a significant shift of the large subunit from the 80S to 60S fractions, while both wild-type and A501U mutant rRNAs were similarly distributed between the 80S and 60S fractions (Supplementary Figure S5).

We next examined the 25S^tag^ rRNA distribution between different ribosome fractions during and after a translational arrest. We shifted exponentially growing cells to dextrose-free medium, followed by dextrose addition back to the culture to re-initiate translation (scheme in Figure 4B and (53)). Aliquots of cells expressing wild-type 25S^tag^ or 25S^tag^-A501U rRNAs were collected, and ribosomal species were resolved by sucrose gradient centrifugation. As expected, the omission of dextrose from medium resulted in a reduction of polysomes and accumulation of 25S^tag^ rRNAs in the 80S monosome fraction (Figure 4B, left and middle panels). Translation was reactivated by 30 min in dextrose-containing medium, as indicated by ribosomes shifting from 80S back to polysomes. The A501U-mutant ribosomes re-populated polysomes as efficiently as wild-type ribosomes (Figure 4B, right panels), suggesting that they are translation initiation-competent. Next, we examined the ability of A501U-ribosomes to participate in translation over time. We shut down the production of new ribosomes in yeast cultures grown in Dox-free media by the addition of Dox and analyzed the distribution of the wild-type and A501U-ribosomes by gradient centrifugation after 0, 2 and 4 h of Dox treatment (Supplementary Figure S6). Gradient analysis showed that similarly to wild-type ribosomes, the A501U-ribosomes were continuously present in the polysomal fractions even after 4 h of transcriptional shut-off (Supplementary Figure S6, right panels). This observation argued against the possibility that the A501U mutation might affect the ribosomes’ participation in new rounds of translation. These data were also consistent with the observed stability of the A501U-ribosomes (Figure 3 and Supplementary Figure S4). Together, the dextrose translation re-initiation (Figure 4B) and Dox shut-off experiments (Supplementary Figure S6) provide strong evidence that ribosomes carrying the A501U mutation are translation initiation-, and likely, elongation-competent. However, some aspects of A501U-mediated translation must be abnormal, as this mutant rRNA cannot support cell viability as the sole source of cellular rRNA in 60S subunits (Figure 2D).

### Ribosomes harboring the A501U mutation induce proteotoxic stress and are sequestered in VHL puncta

**Figure 5.**
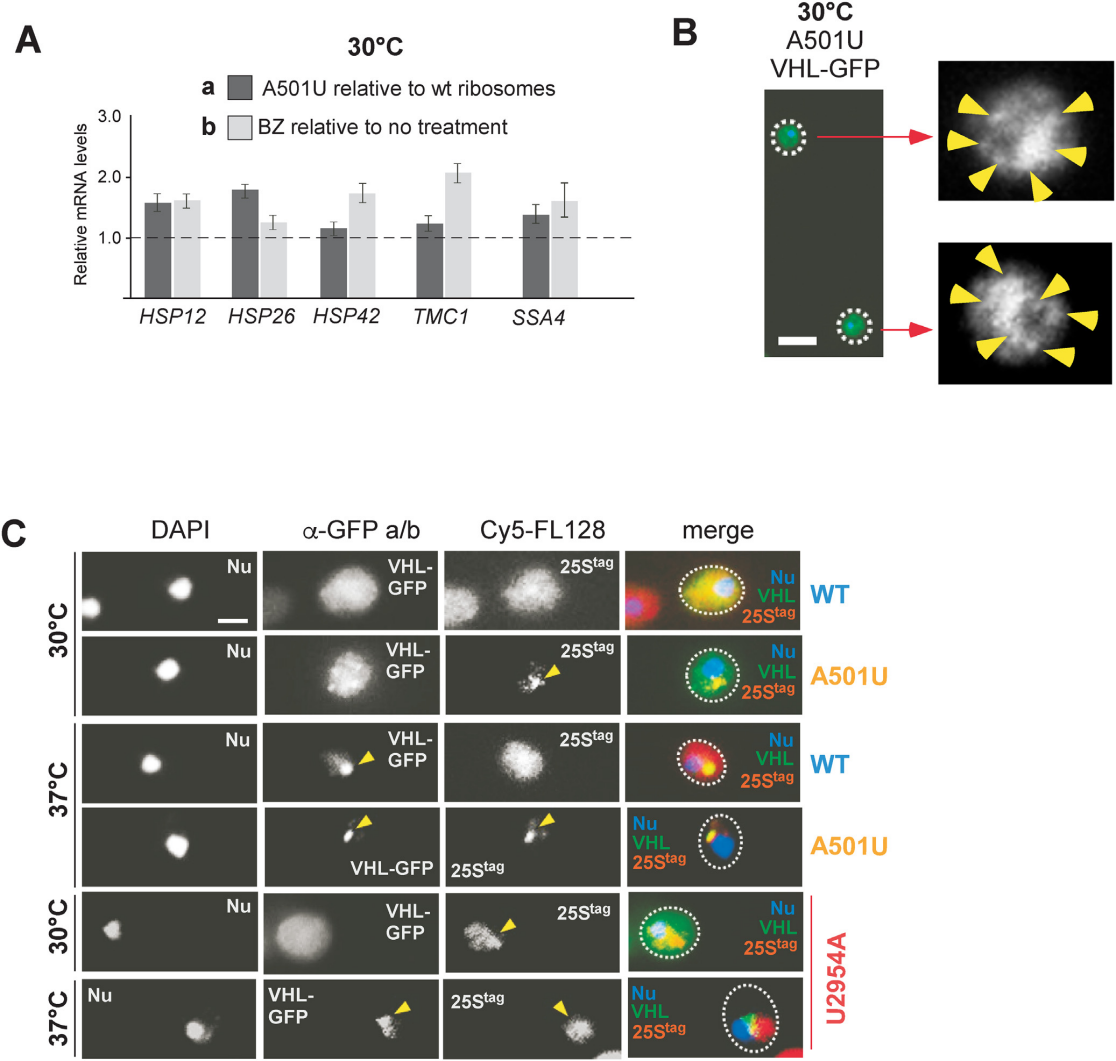
25S^tag^-A501U rRNA induces proteotoxic stress and colocalizes with VHL puncta. (**A**) mRNA quantified by RT-qPCR relative to the *ACT1* mRNA. Dark grey bars, BY4741 cells transformed with pDP333 carrying wild-type 25S^tag^ or 25S^tag^-A501U rRNAs were grown in the absence of Dox. Light grey bars, BY4741 expressing wild-type 25S^tag^ rRNA were treated with 50 μM Bortezomib (BZ) for 1 h. Graph shows mean values normalized to the 25^tag^-wt rRNA in untreated cells (dashed horizontal line, Y = 1) in triplicate samples, error bars, S.D. (**B**) BY4741 cells expressing 25S^tag^-A501U rRNA and VHL-GFP were grown at 30°C. Yellow arrows point to VHL puncta detected by indirect immunofluorescence staining with anti-GFP and Alexa 488-conjugated antibodies. Scale bar, 5 μm. (**C**) BY4741 cells expressing wild-type 25S^tag^, 25S^tag^-A501U or 25S^tag^-U2954A rRNAs and VHL-GFP were shifted to 30°C or 37°C for 30 min. VHL-GFP was visualized by immunofluorescence staining as in (B), while 25S^tag^ rRNAs were detected by RNA-FISH using probe Cy5-FL128. DAPI was used to stain the nucleus (Nu). Representative microscopy fields are shown. Scale bar, 2 μm.

In general, translational abnormalities might manifest as error-prone translation, i.e. generation of abnormal proteins (54). It seemed possible that A501U-ribosomes might generate a subset of aberrant polypeptides due to, for example, altered rate of translation (55), poor decoding capacity triggered by the mutation in ES7L (56), or failure to interact with co-translational chaperone(s) (57,58) leading to a peptide misfolding. In this case, cells exposed to translation by A501U-ribosomes should experience proteotoxic stress. To address this possibility, we first measured levels of proteotoxic stress markers in cells expressing 25S^tag^-A501U rRNA in comparison to wild-type 25S^tag^ rRNA at 30°C (Figure 5A-a). Separately, we treated wild-type 25S^tag^ rRNA-expressing cells with bortezomib (BZ) to induce proteotoxic stress by an alternative mechanism (59). BZ is an efficient inhibitor of the chymotrypsin-like activity of the proteasome (60) and has been successfully used in yeast (7). We examined the expression of the same proteotoxic stress-responsive genes at 30°C in BZ-treated cells relative to cells that were not treated with the drug (Figure 5A-b). The RT-qPCR analysis showed that expression of A501U-ribosomes promoted upregulation of *HSP26, HSP12, HSP42, TMC1* and *SSA4* mRNAs to an extent similar to that after treatment of wild-type 25S^tag^ rRNA expressing cells with BZ (Figure 5A). These results suggest that A501U-ribosomes induce proteotoxic stress on a level similar to that occurring after the inhibition of the proteasome, supporting the idea that translational abnormalities associated with A501U-ribosomes might be linked to the production of aberrant polypeptides.

As noted in the Introduction, proteotoxic stress often causes the formation of protein inclusions. To determine whether A501U-ribosomes might promote sequestration of misfolded cytoplasmic proteins into puncta or foci structures (61), we first examined the distribution of the misfolded marker protein VHL-GFP in cells expressing 25S^tag^-A501U rRNAs using indirect immunofluorescence. VHL (von Hippel-Lindau) is a tumor suppressor protein that adapts its final, proper conformation upon binding to the cofactor elongin BC (62). When expressed in yeast that lack elongin, VHL remains in an unfolded state and in response to heat shock (37°C) forms inclusions in the vicinity of the nucleus termed JUNQ (5). Remarkably, upon 25S^tag^-A501U rRNA expression, we detected the formation of multiple small-sized VHL-GFP puncta distributed throughout the cytoplasm at 30°C (Figure 5B). The GFP-antibody used in this experiment showed no non-specific signal in cells transformed with the empty vector control (Supplementary Figure S7A). Given that sequestration of proteins into cytoplasmic inclusions is indicative of proteotoxic stress (11), and in agreement with the RT-qPCR data (Figure 5A), we concluded that the A501U-ribosomes alone may promote proteotoxic stress in cells at the normal growth temperature of 30°C.

To further corroborate these data, we examined VHL-GFP distribution in the cytoplasm under more diverse conditions. As such, we tested cells expressing 25S^tag^-A501U and wild-type 25S^tag^ rRNAs at two different temperatures, 30°C and 37°C. In agreement with a previous study (5), at 37°C, VHL-GFP was sequestered into a single cytosolic inclusion regardless of the type of tagged ribosomes present, whereas at 30°C, VHL-GFP was distributed evenly throughout the cytoplasm in cells expressing wild-type ribosomes and became sequestered into multiple small foci in cells expressing mutant ribosomes (Figure 5C, the α-GFP a/b column), consistent with the previous data (Figure 5B). To confirm the expression of tagged ribosomes in these cells, we combined immunofluorescence with fluorescent in situ hybridization (FISH). We used the fluorescent probe against the 25S rRNA tag (Cy5-FL128). The A501U-mutant, but not wild-type, tagged 25S rRNA formed foci (hereafter, the ribosome-enriched foci) at both temperatures tested (Figure 5C, A501U rows). Strikingly, the A501U-ribosome-enriched foci, but not the 25S-NRD substrate rRNA-U2954A, colocalized with the JUNQ marker VHL, notably evident at 37°C (Figure 5C and Supplementary Figure S8A). The A501U-ribosome-enriched foci showed no co-localization with Htt103QP (Supplementary Figure S8B), which we used here as a control due to its ability to form a different type of cytosolic inclusions containing insoluble aggregates (63).

Taken together, these results demonstrate that (i) A501U-mutant ribosomes are capable of inducing proteotoxic stress, and (ii) A501U-mutant 60S particles are targeted into the same cellular sites where misfolded soluble proteins are captured.

### 25S^tag^-A501U rRNA, but not 25S^tag^-U502A or 18S^tag^ rRNAs, is sequestered into ribosome-enriched foci

**Figure 6.**
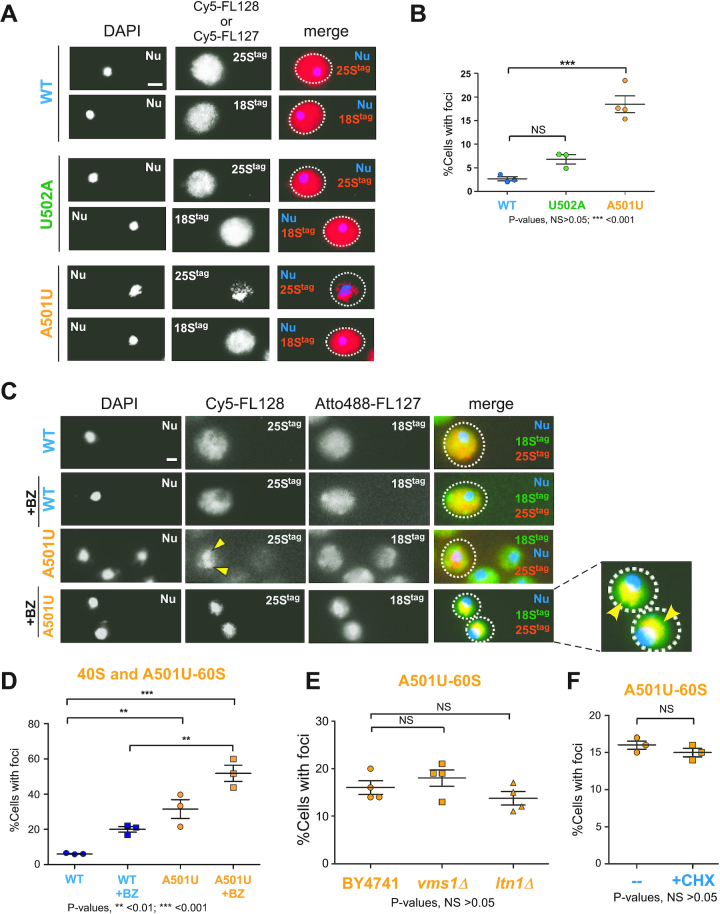
Sequestration into ribosome-enriched foci involves both 40S and 60S-A501U subunits and is not a part of 60S-RQC. (**A**) BY4741 cells expressing indicated 25S^tag^ rRNAs were analyzed by RNA-FISH using probes against 18S^tag^ (Cy5-FL127) and 25S^tag^ (Cy5-FL128) rRNAs. Scale bar, 2 μm. (**B**) Percentage of cells with 25S^tag^ rRNA foci in cell populations transformed with the indicated mutant 25S rRNA constructs (*n* > 100 cells). Mean values and standard errors are shown, P values were determined by one-way ANOVA plus Dunnett's multiple comparisons test, NS is non-significant. (**C**) Cells were grown as in (A), and half of each culture was treated with 50 μM Bortezomib (BZ) for 1 h. 25S^tag^ rRNA was visualized by FISH with the Cy5-FL128 probe, while 18S^tag^ rRNA was visualized with the Atto 488-FL127 probe. An enlarged image for 18S^tag^ and 25S^tag^-A501U rRNAs co-staining after BZ treatment is shown on the right. Yellow arrows point to the A501U-ribosome enriched foci. DAPI staining visualizes the nucleus (Nu). Representative microscopy fields are shown; scale bar, 2 μm. (**D**) Percentage of cells with 25S^tag^ rRNA foci in cell populations expressing the indicated 25S^tag^ rRNA constructs treated or untreated with BZ for 1 h (*n* > 100 cells). Mean values and standard errors are shown, P values were determined by one-way ANOVA plus Dunnett's multiple comparisons test. (**E**) pDP333 containing 25S^tag^-A501U rRNA was transformed into wild-type BY4741, *ltn1Δ* and *vms1Δ* strains; transformants were grown as in (A), cells were analyzed by FISH with the Cy5-FL128 probe. Percentages of cell populations displaying 25S^tag^ rRNA foci were quantified for each strain tested (n>200 cells). Mean values and standard errors are shown, P values were determined by one-way ANOVA plus Dunnett's multiple comparisons test, NS is non-significant. (**F**) BY4741 cells containing 25S^tag^-A501U rRNA were grown as in (A), one half of this culture was treated with 50 μg/ml cycloheximide (CHX) for 1 h prior to FISH with the Cy5-FL128 probe. Cells containing foci were quantified as in (E), >200 cells were analyzed. P value was determined by Mann Whitney test, NS is non-significant.

Next, we characterized the ribosome-enriched foci of misfolded soluble proteins in detail. We used FISH with Cy5-labeled FL127 probes to detect 18S and with FL128 probes to detect 25S rRNA tags. To verify that fluorescent signal derived from hybridizations with these probes is rRNA-tag-specific, we hybridized cells harboring an empty vector control with Cy5-FL127 and Cy5-FL128. We detected no fluorescent signal (Supplementary Figure S7B & C), confirming the lack of unspecific cross-reactivity with endogenous RNA or DNA. Consistent with previous experiments (Figure 5C), FISH analysis of cells expressing wild-type 25S^tag^ and 18S^tag^ rRNAs showed strong fluorescent signal that in the vast majority of cells was distributed throughout the cytoplasm for both tags (Figure 6A, top panels). Similar to wild-type rRNAs, the fluorescent signal derived from 18S^tag^ and 25S^tag^-U502A rRNAs was uniformly spread (Figure 6A, middle panels). In contrast, the A501U mutation affected the uniform intracellular 25S^tag^ rRNA distribution (Figure 6A, bottom panels) with about one out of five cells (Figure 6B) demonstrating 25S^tag^-A501U rRNA in ribosome-enriched foci localized in the vicinity of the nucleus, while 18S^tag^ rRNA retained diffused cytoplasmic localization (Figure 6A, bottom panels). Although Cy5-FL128-positive foci were also detectable in a small percentage of cells expressing wild-type or U502A mutant ribosomes (∼2% or ∼7%, respectively, Figure 6B), the A501U mutation significantly increased the number of cells with the ribosome-enriched foci (∼20%, Figure 6B).

Multiple types of ribosome biogenesis defects can result in a failure of the nuclear export of nascent ribosomal particles (reviewed in (64)). To rule out the possibility that 25S^tag^-A501U rRNA-containing ribosome-enriched foci might represent accumulation of immature 60S subunits trapped in the nucleus, we combined FISH with immunostaining for the nucleolar marker Nop1. This dual staining revealed no overlap of fluorescent signals derived from 25S^tag^-A501U rRNA and Nop1 (Supplementary Figure S8C), confirming that the newly identified foci are cytosolic. In another control experiment, we co-stained ribosome-enriched foci with the P-body marker Dcp2-GFP after shifting yeast culture to dextrose-free medium for 30 min to promote P-body formation (65). The rationale for this control experiment was that 18S rRNA containing a deleterious mutation in the DCS was previously detected in P-bodies (19). However, no overlap between 25S^tag^-A501 rRNA and Dcp2-GFP signals was observed (Supplementary Figure S8D). Thus, the A501U mutation in the ES7L region of 25S rRNA results in the formation of translationally active ribosomes that poorly perform during translation causing proteotoxic stress and co-localize with cytosolic misfolded soluble proteins.

### Both 40S and 60S-A501U ribosomal subunits can be detected in ribosome-enriched foci

Yeast cytoplasmic sites of VHL accumulation are thought to represent compartments for the triage, refolding, and degradation of misfolded proteins (5). The ability of A501U-ribosomes to participate in translation along with their failure to support growth in NOY891 cells (Figure 2D) and the induction of proteotoxic stress in BY4741 cells (Figure 5A and B) led us to consider a model in which A501U-ribosomes synthesize aberrant polypeptides, facilitating transport of either mutant 60S subunits or mutant 80S ribosomes into the VHL-containing foci.

To distinguish between these possibilities, we first asked whether 40S subunits are present in the A501U-ribosome enriched foci by using dual-probe FISH analysis with probes Cy5-FL128 against the 25S rRNA and Atto 488-FL127 against 18S rRNA. Similar to Cy5-FL127 and Cy5-FL128, the Atto 488-FL127 probe showed no detectable background hybridization with cellular RNA or DNA in the empty vector control strain (Supplementary Figure S7B). We reasoned that if the entire ribosome bound to a nascent polypeptide is translocated to the ribosome-enriched foci, 40S would be present in the same foci as 60S-A501U. Because the 60S-A501U subunits represent only a minor fraction of the total 60S pool and they can pair with any available (tagged and untagged) 40S during initiation, sequestration of the 40S subunits would be difficult to assess by FISH with an 18S^tag^ probe under regular culture conditions. Indeed, when cells expressing 25S^tag^ and 18S^tag^ rRNAs were hybridized with Cy5-FL128 and Atto488-FL127 probes, the 18S^tag^ rRNA-derived Atto488 signal was distributed throughout the cytoplasm irrespective of whether 25S^tag^-A501U or wild-type 25S^tag^ was expressed in the cells (Figure 6C). Considering the dynamic nature of the VHL puncta/JUNQ and the dependence of their disaggregation on the ubiquitin-proteasome system (UPS) (5,7), we asked whether inhibition of proteasome activity would stabilize 40S subunits in the A501U-ribosome enriched foci. To test this hypothesis, we treated cells expressing 25S^tag^-A501U rRNA with BZ prior to dual-probe FISH analysis. We found that a short BZ treatment resulted in an almost complete colocalization of tagged 40S subunits with 60S subunits in the cytoplasmic foci, especially notable for A501U-ribosomes (Figure 6C enlarged image, and Figure 6D), supporting the hypothesis that entire ribosomes are captured in cytoplasmic regions containing soluble misfolded proteins.

### The A501U-ribosomes are not substrates of ribosome-associated quality control (RQC)

To gain further insights into the mechanism of the A501U-ribosome-enriched foci formation, we examined the participation of the ribosome-associated quality control system (RQC, (66)) in this process. Recent work by the Inada group has demonstrated that 18S-NRD employs the RQC machinery to split DCS-mutated terminally stalled ribosomes into subunits, liberating the non-functional 40S for degradation (22). This finding is in accord with a study showing that ribosomes that contain immature 60S particles that retain ITS2 sequences in their 25S rRNA are recognized by cytosolic RQC factors, including Ltn1, after becoming stalled on mRNA (67). Following these leads, we tested the distribution of 25S^tag^-A501U rRNA in cells deleted for two major components of RQC, the E3 ubiquitin ligase Ltn1 (68) and the peptidyl-tRNA processing factor Vms1 (69,70). However, we found no significant changes in the fraction of cells containing the A501U-ribosome-enriched foci in *ltn1Δ* and *vms1Δ* strains compared to the wild-type strain (Figure 6E), indicating that the formation of these foci is not a part of the Ltn1- or Vms1-dependent RQC. Given that RQC operates predominantly on stalled ribosomes (66), we next enforced ribosome stalling by treating cells with the translational inhibitor cycloheximide (CHX) and evaluated ribosome-enriched foci in drug-treated and untreated cells. Consistent with results obtained with RQC mutants (Figure 6E), CHX did not affect the number of cells with ribosome-enriched foci (Figure 6F), further supporting that A501U-ribosomes are not RQC substrates.

### Sequestration of ribosomes into the ribosome-enriched foci is a general response to proteotoxic stress

**Figure 7.**
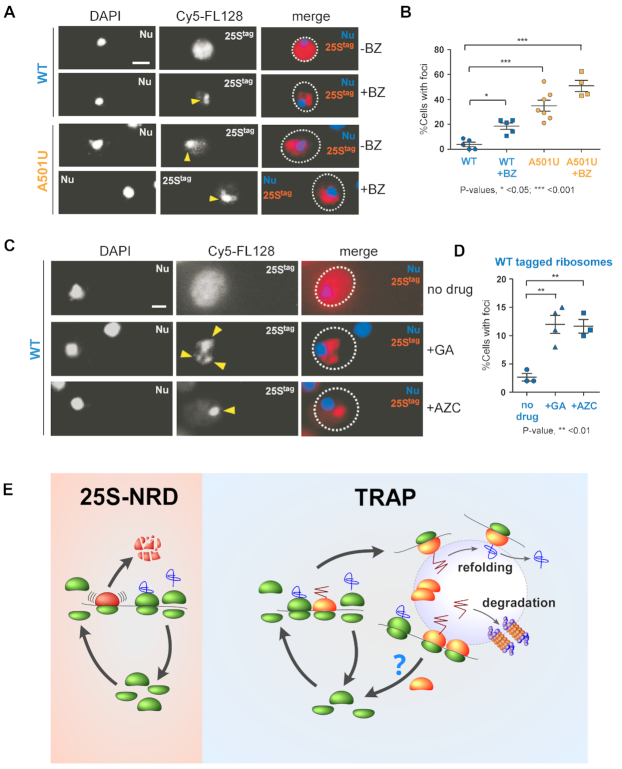
Wild-type ribosomes are sequestered into ribosome-enriched foci during proteotoxic stress. (**A**) BY4741 cells expressing wild-type 25S^tag^ or 25S^tag^-A501U were grown in the absence of Dox and then treated with 50 μM Bortezomib (BZ) for 1 h or remained untreated. 25S^tag^ rRNAs were detected with the Cy5-FL128 probe. Yellow arrows point to ribosome-enriched foci, DAPI staining visualizes the nucleus (Nu). Representative microscopy fields are shown. Scale bar, 2 μm. (**B**) Percentages of the analyzed cell population (*n* > 100 cells) containing ribosome-enriched foci were calculated. Bars show mean values and standard errors. The data are representative of three independent repeats. P values were determined by one-way ANOVA plus Dunnett's multiple comparisons test. (**C**) BY4741 cells transformed with pDP333 carrying wild-type 25S^tag^ rRNA were grown in the absence of Dox, and then either treated with 50 μM geldanamycin (GA) or 50 μM azetidine-2-carboxylic acid (AZC) for 1 h or remained untreated. 25S^tag^ rRNAs was detected with the Cy5-FL128 probe. Yellow arrows point to the ribosome-enriched foci. (**D**) Cell containing foci were quantified as in (B). P values were determined by one-way ANOVA plus Dunnett's multiple comparisons test. (**E**) Working model. *Left:* During 25S-NRD, the 60S subunit containing mutations inactivating the PTC undergoes stalling, followed by the release and degradation of the dysfunctional subunit. *Right:* Ribosomes that generate anomalous nascent polypeptide chains (red zig-zag) are subject to Translational Relocalization with Aberrant Polypeptides (TRAP), upon which mistranslated polypeptides sequester the entire malfunctioning ribosomes into cellular compartments where the nascent chains are refolded or degraded if refolding fails.

Does sequestration of ribosomes into cytoplasmic foci represent a general cellular response? To address this question, we analyzed the distribution of the wild-type 25S^tag^ rRNA in cells treated with BZ. We found that BZ increased sequestration of wild-type tagged ribosomes into cytoplasmic foci (∼20% cells, Figure 7A and B) to a level similar to that of cells expressing A501U-ribosomes without any drug-treatment (compare Figures 6B third column and 7B second column). As expected, BZ further increased the number of cells containing ribosomal foci in the 25S^tag^-A501U rRNA expressing culture (up to ∼50%, Figure 7A and B), likely due to synergistic effects of mistranslation by the mutant ribosomes and the aberrant peptide accumulation due to BZ-dependent degradation defects. To corroborate this result, we induced the production of aberrant polypeptides by alternative means and quantified foci-containing cells in the wild-type 25S^tag^ rRNA-expressing culture. We found that inhibition of the Hsp90 chaperone with geldanamycin (71) or treatment of cells with the toxic proline analog azetidine-2-carboxylate (AZC), which induces polypeptide misfolding (72), also led to increased relocalization of the wild-type ribosomes into foci (Figure 7C and D). These results suggest that the sequestration of ribosomes into cytosolic protein foci is not limited to A501-mutant ribosomes but likely represents a general cellular strategy to deal with abnormal polypeptides and ribosomes that generate them.

## DISCUSSION

We generated eight mutants of the yeast ribosome by introducing single or double-base substitutions into ES7L - an enigmatic region in 25S rRNA located on the surface of the 60S ribosomal subunit. Mutations in the evolutionarily conserved central junction of ES7L (ES7L_CJ_, Figure 1A) resulted in only slight alterations in the ES7L structure (Figure 1B). However, seven out of eight of these mutations were lethal (Figure 2D). The viable U502A and the nonviable A501U mutations were selected for further analysis based on their minor structural but strong phenotypical difference (Figures 1B and 2D). We found that the nonviable A501U mutation did not affect 25S rRNA stability (Figure 3 and Supplementary Figure S4) or 25S rRNA abundance in ribosomes among translating polysomes (Figure 4 and Supplementary Figures S5 and S6). However, A501U caused the sequestration of ribosomes into cytosolic compartments shared with misfolded soluble proteins (Figure 5C). By contrast, the viable U502A mutation did not cause sequestration of ribosomes into cytosolic compartments.

The sequestration of mutated ribosomal subunits into specific cellular sites has been reported previously for NRD substrates (19). As such, the 18S-NRD substrates are captured into P-bodies, similar to aberrant mRNAs in the NGD (No-Go-Decay) pathway (19), presumably to facilitate their degradation via the enzymatic machinery present within these bodies (73). We suspected that the A501U-ribosomes might represent a novel NRD substrate; however, four lines of experimental evidence strongly argue against this possibility. First, 25S^tag^-A501U rRNA was found to have significantly greater lifetime than the known 25S-NRD substrate 25S^tag^-U2954A rRNA, which decays quickly (Figure 3 and Supplementary Figure S4). Second, the A501U ribosomes coalesce into distinct cytoplasmic compartments shared with misfolded proteins, whereas U2954A mutant ribosomes do not (Figure 5C and Supplementary Figure S8A). Third, unlike NRD substrates, the A501U-mutant ribosomes were found to escape the RQC surveillance pathway (Figure 6E and F), which rapidly eliminates aberrant polypeptides associated with ribosomes of NRD pathway to prevent proteotoxic stress (22,66). Fourth, a double-probe FISH experiment detected the complete colocalization of wild-type 40S and mutant 60S-A501U subunits in cytoplasmic foci (Figure 6C), suggesting the possibility that polysomes become sequestered. This is in contrast to NRD substrates, which represent single subunits formed prior to their sequestration and degradation through the resolution of terminal translational stalls (19,22).

The most remarkable finding of this study was that the A501U-ribosomes become sequestered into cytoplasmic inclusions shared with misfolded protein (Figure 5C), a phenomenon that has not been reported previously. Although direct evidence is lacking, the data are consistent with a model in which the unicellular eukaryote Saccharomyces cerevisiae possesses a previously unrecognized pathway to sequester malfunctional ribosomes together with aberrant polypeptides into specialized cytoplasmic foci that accumulate misfolded soluble proteins (Figure 7E). We will refer to this mechanism as TRAP (Translational Relocalization with Aberrant Polypeptides). We propose that during TRAP (see detailed scheme on Figure 7E, right), aberrant nascent chains generated by faulty ribosomes serve as sequestration tags and promote relocalization of translating ribosome complexes. Inside the protein inclusions, the aberrant polypeptides undergo triage followed by their refolding or degradation. We also found that TRAP operates with non-mutated ribosomes; protein misfolding induced by various drugs resulted in the sequestration of wild-type ribosomes into the same cytosolic foci (Figure 7A and D).

One currently lacks technical tools to discern the translational products of the A501U-ribosomes because cells that fully rely on them (in the NOY891 strain) are not viable (Figure 2D), and upon expression in the wild-type strain the A501U-ribosomes constitute a small portion of the total ribosomal pool (Supplementary Figures S1 and S2). However, low translational fidelity has been observed previously for ribosomes with mutations in their auxiliary regions. Recently published work has shown that ribosomes containing partial non-lethal deletion of the expansion segment 27L (ES27L) promote frameshifting, stop codon readthrough and amino acid mis-incorporation, presumably by affecting precise positioning of the key enzymes responsible for nascent chain modifications and processing (28). In line with this study, it was recently found that cells lacking the ribosome-associated chaperone Ssb/RAC, which directly interacts with nascent chains and participates in newly generated polypeptide folding (74), also exhibit low fidelity of translation (58). It is also important to mention that a biochemical pulldown using synthetic ES7L has identified a group of chaperones that may potentially interact with ES7L (56), raising the possibility that the A501U mutation might disturb the chaperone-assisted, co-translational folding of the nascent polypeptide chains in analogy with the Ssb/RAC chaperone model (58), thus, generating abnormal polypeptides.

Although the extent of A501U-ribosome incorporation into polysomes was comparable with that of wild-type ribosomes (Figure 4 and Supplementary Figures S5 and S6), we currently cannot assess efficiency and/or accuracy of A501U-ribosome translation elongation and termination. In analogy with other known translational defects, A501U-ribosomes may affect translation elongation and/or termination in many ways. As such, the A501U mutation may disturb optimal speed of translation, affecting accuracy of decoding (55), force ribosomes to operate unconventionally on translation-challenging sequences or stop codons (58), indirectly change codon selection (75), induce transient translational pausing (76) or promote premature folding of nascent peptide domains (57), ultimately causing production of aberrant proteins.

We have not determined the fate of entrapped ribosomes, which would require development of microscopy-based single cell life-imaging platform. Thus, the question on whether ribosome entrapment is permanent or transient remains open. According to the permanent TRAP model, detaining the ribosomes inside the foci, while maintaining their stability (Figure 3D and Supplementary Figure S4), might play a role of *isolation strategy*, so that wild-type ribosomes will not be mixed with malfunctional ribosomes. Under this scenario, TRAP can be rated as a *true ribosome surveillance pathway*, which, however, differs from NRD by protecting ribosomes from degradation and by preventing them from participating in new rounds of translation. How long entrapped ribosomes remain in the cytoplasmic structures and which cellular mechanisms are involved in their clearance awaits further experimentation.

Based on an alternative, transient TRAP model, ribosomes are liberated and return to active translation after delivering aberrant polypeptides to a site of triage and protein QC. Under this hypothetical scenario, TRAP does not operate as a mechanism of ribosome surveillance *per se*, but rather functions primarily as a protein-quality control tool. However, compared to wild-type 40S subunits that are released from cytoplasmic foci quickly (Figure 6C), mutant 60S subunits may spend longer time in these compartments (Figures 5C, 6A and C), and are temporarily absent from the active translational pool. Therefore, one could also view TRAP as a *passive* ribosome-surveillance pathway, which operates by mitigating detrimental effects from the defective ribosomes lacking obvious features that would trigger their degradation. This unusual model implies that it might simply be more economically profitable for a cell to eliminate faulty translation products than to thoroughly ‘debug’ the machinery that produces them. In fact, aside from mutations in ES7L, faults in various ribosome functions may arise from diverse genetic mutations or damage to multiple sites on rRNA or r-proteins occurring during the ribosome's lifespan. It is difficult to imagine cells developing specialized pathways for the detection and elimination of every possible mutation conferring translational defects. Considering that eukaryotic cells possess a plethora of protein quality control pathways that operate co- and post-translationally (11,77,78), a more parsimonious solution seems to outsource problematic translational products to these already existing systems rather than build new surveillance machineries. Thus, transient TRAP may represents a cost-effective way for the eukaryotic cell to manage resources by exploiting the cooperativity between protein quality control and translation systems.

Our results revealed several important characteristics of ribosomes carrying the A501U mutation in the ES7L: A501U-ribosomes (i) escape NRD-dependent degradation and remain intact; (ii) are active in translation but possess a functional defect that leads to cell lethality if not compensated by wild-type ribosomes and (iii) are moved to cytoplasmic foci that accumulate misfolded proteins. These observations corroborate non-deleterious nature of the A501U mutation. Why then do A501U-ribosomes not support cell growth in the absence of endogenous ribosomes (Figure 2D)? One explanation might be that the A501U mutation changes ribosomal preference for mRNAs, resulting in translation of only subset of transcripts excluding essential genes and, thus, conferring lethality when expressed in the NOY891 strain. It is also possible that the genetically modified NOY891 strain might be sensitive to even low-dose proteotoxic stress caused by A501U-ribosomes (Figure 5A), or it might be defective in PQC.

This study opens new attractive avenues for investigation. For example, it is important to better understand the formation, structure and dynamics of TRAP centers. As such, it is possible that the JUNQ/VHL puncta (where A501U-ribosomes localize) represents self-organizing droplets in a liquid-like state (79–81) and may thus concentrate misfolded proteins from different origins, possibly including TRAP-substrates (Figure 5C). It would be interesting to see if ribosomes themselves might play a role in the phase separation process. Given that yeast ribosomes subjected to TRAP carry a mutation in the evolutionarily conserved core of ES7L (Figure 1A), it is possible that similar mutations in human ribosomes might also lead to TRAP. This line of investigation could provide valuable new insights into proteostasis defects such as those in neurodegenerative diseases, and aid in designing new pharmacological approaches for their treatment.

## Supplementary Material

gkaa068_Supplemental_File
