# Supplementary material for: Proteotoxic stress promotes entrapment of ribosomes and misfolded proteins in a shared cytosolic compartment
Source: Nucleic Acids Res. 2020 Feb 7;48(7):3888–905. doi: 10.1093/nar/gkaa068 (PMC7144922; doi:10.1093/nar/gkaa068)
Supplement: gkaa068_Supplemental_File [file gkaa068_supplemental_file.pdf]

SUPPLEMENTAL DATA

Table S1. Oligonucleotides used for rRNA and snoRNA analysis.

Name (probe, primer)	Sequence	Application
y520	5′TTGGATGTGGTAGCCGTT-3′	Northern to detect endogenous 18S rRNA
y540	5′-TCCTACCTGATTTGAGGTCAAAC-3′	Northern to detect endogenous 25S rRNA
FL127	5′-GCCGAGGATCCAACTAGGGGGCT-3′,	Northern and FISH to detect tagged 18S rRNA
FL128	5′-GGGCAGGCTGCAGCTTCCTACCAG-3′	Northern and FISH to detect tagged 25S rRNA
U3	5′-ACCAAGTTGGATTCAGTGGC-3′	Northern to detect U3 snoRNA
25S-A	5'-AAGAAACCAACCGGGATTGC-3'	PCR analysis of rRNA content
25S-B	5'-ATTACAACTCGGGCACCGAAG-3'	PCR analysis of rRNA content
FL128-B	5'-GGGCAGGCTGCAGCTTCC-3'	PCR analysis of rRNA content
18S-C	5'-AAAATCAATGTCTTCGGACTCT-3'	PCR analysis of rRNA content
18S-D	5'-TCGAAAGTTGATAGGGCAGAA-3'	PCR analysis of rRNA content
FL127-C	5'-CCCTAGTTGGATCCTCGGC-3'	PCR analysis of rRNA content

Table S2. Plasmid constructs used in this study.

Plasmid	Source	Reference	Manipulations
pJD694	John Dinman	(1)	See text
pWL160	Melissa Moore	(2)	See text
pDP333	This study	none	See text
pNOY353	Masayasu Nomura	(3)	none
pYes-Htt103QP	Yury Chernoff	(4)	none

pRS313-Dcp2-GFP	This study		Dcp2-GFP fragment was excised with Xba I from pRP1175-DCP2-GFP and cloned into pRS313 at the Xba I site
pRP1175-DCP2-GFP	Roy Parker	(5)	
PESC-LEU-GFP-VHL	Judith Frydman	Addgene #21053	
PESC-URA-GFP-VHL	This study		PESC-LEU-GFP-VHL was digested with Sac I and Nhe I. The 1.5 kb fragment was cloned into PESC-URA between its Sal I and Nhe I sites.
pESC-LEU	Stratagene, #217452		
PUAD	Randy Strich	(6)	
pNS1	This study		PESC-LEU was digested with Not I and Xho I. The plasmid backbone was ligated with the Not I / Xho I fragment containing the ADH1 promoter that was excised from PUAD.

Supplemental Figure 1

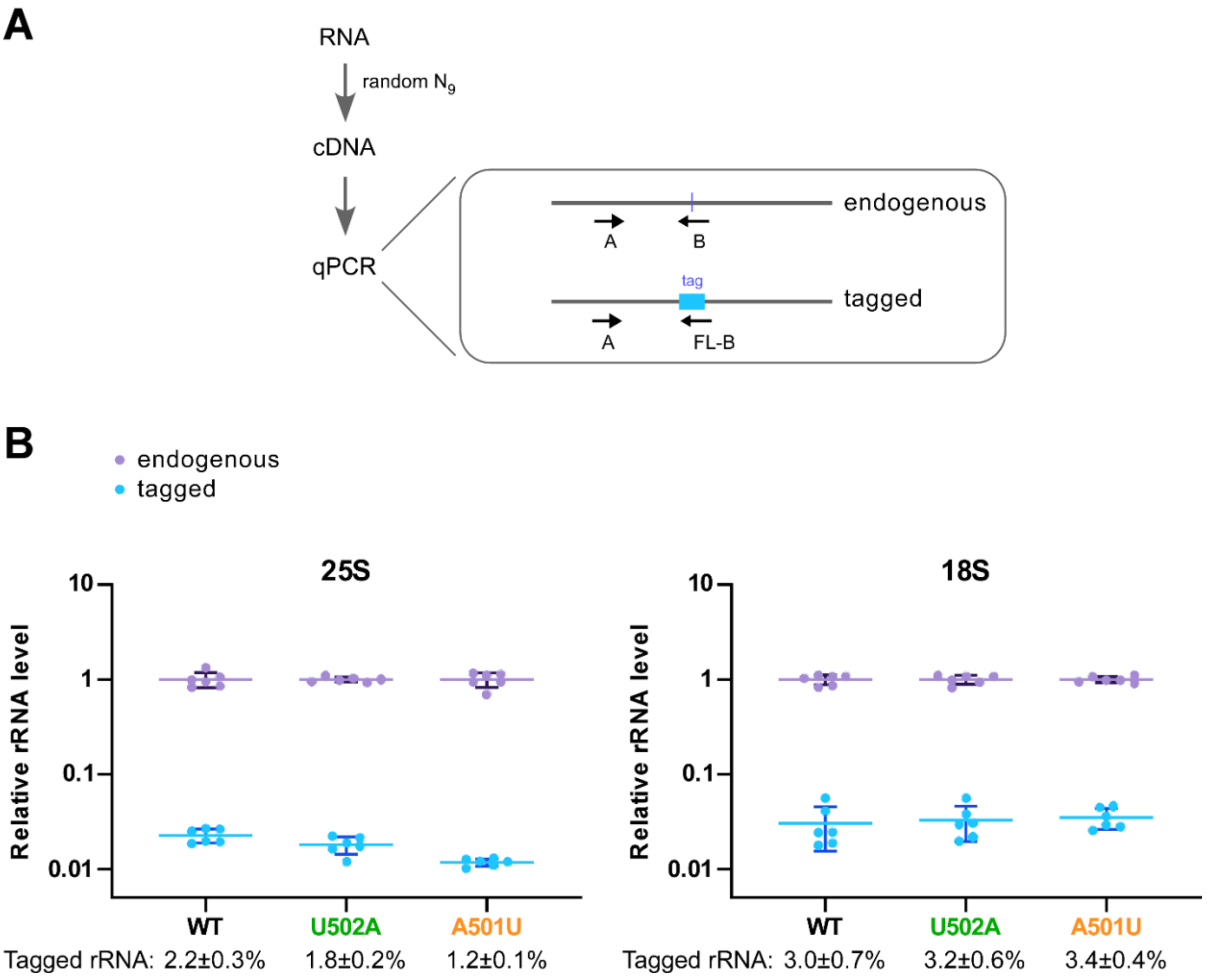

Figure S1. Quantification of the relative levels of the endogenous and pDP333-derived rRNAs. (A) Outline of the experimental procedure. Primer combinations A+B and A+FL-B amplify only endogenous (untagged) or plasmid-expressed (tagged) 25S rRNAs. Analogous primer combinations were devised for 18S rRNAs. Primer sequences are provided in Supplemental Table S1. **(B)** Relative levels of rRNAs normalized to average endogenous rRNA levels. For each strain expressing the indicated wild-type or mutant rRNA, two individual colonies were picked and analyzed in three technical replicates. Molar ratios were calculated from the RT-qPCR data; see Supplemental Methods for complete experimental details. Graphs show individual data points and average; error bars, S.D; n=6. The calculated average percentage values and S.E.M. are shown below the graphs for each strain

Supplemental Figure 2

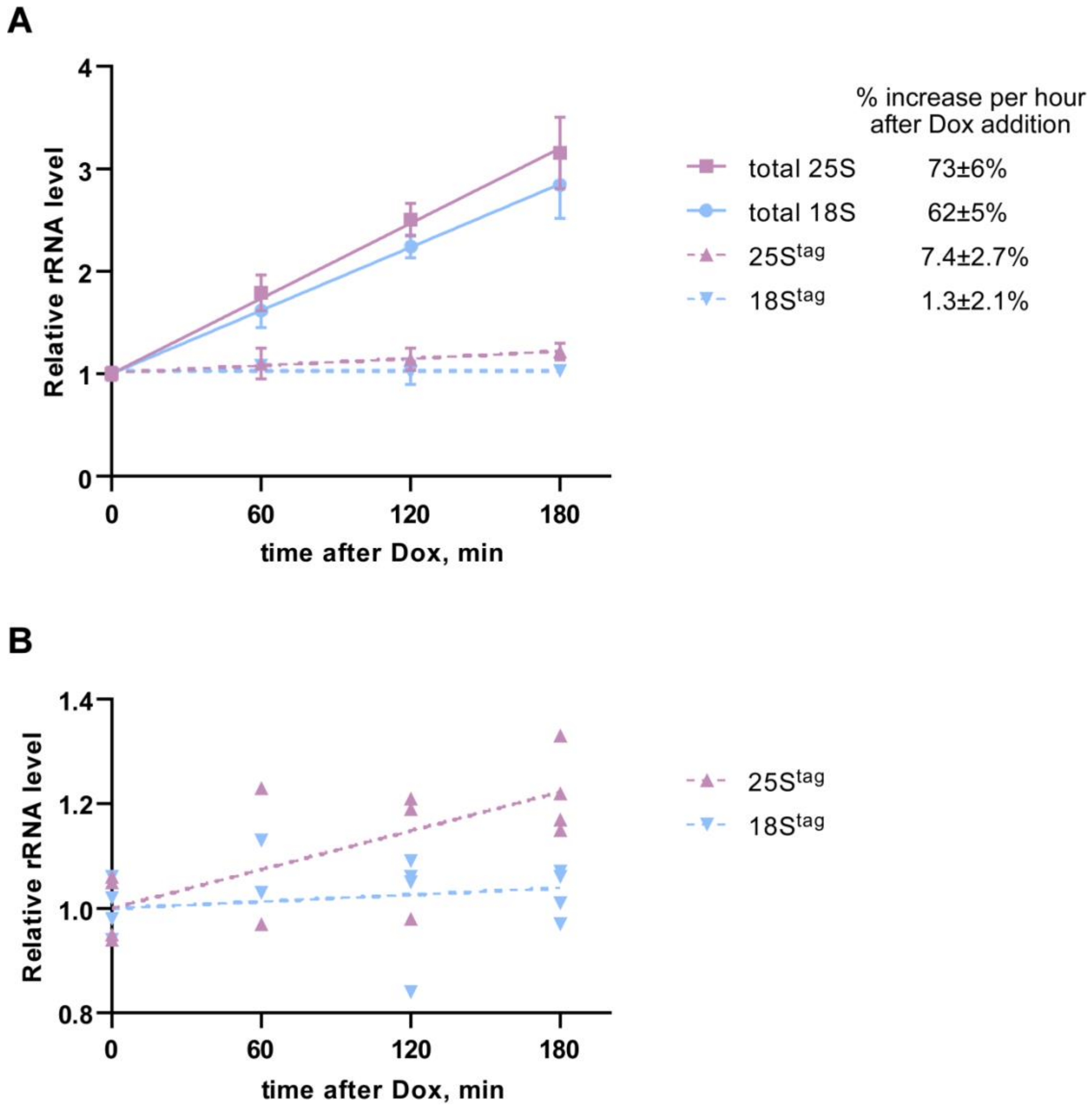

Figure S2. Accumulation of endogenous and plasmid-derived tagged rRNAs following Dox addition. Cells were grown overnight in Dox-free synthetic medium at 30°C to generate tagged rRNAs. The overnight culture was diluted to an OD_{600} of ~0.35 with fresh medium to start four replicate cultures. Dox was added to 10 μg/ml when these cultures reached the OD_{600} of 0.7, and samples were then withdrawn from the cultures every hour. RNA from equal culture volumes was analyzed by Northern hybridizations to quantify the amounts of total and tagged rRNAs with the rRNA- or tag-specific probes (Supplemental Figure S1) at each time point. (**A**) Relative increases of tagged and total rRNAs after Dox addition. The rate of increase per hour (±95% C.I.)

was estimated by linear regression curve fitting. The graph in (**B**) shows the tagged rRNA data in a different scale to better visualize the dynamics of the tagged rRNA after Dox addition. In the presence of Dox, $25S^{tag}$ continues to accumulate with a low rate slightly ahead of $18S^{tag}$, likely reflecting the leakiness of the Tet-regulated promoter and differential stability of the nascent rRNAs in this system.

Supplemental Figure 3

A

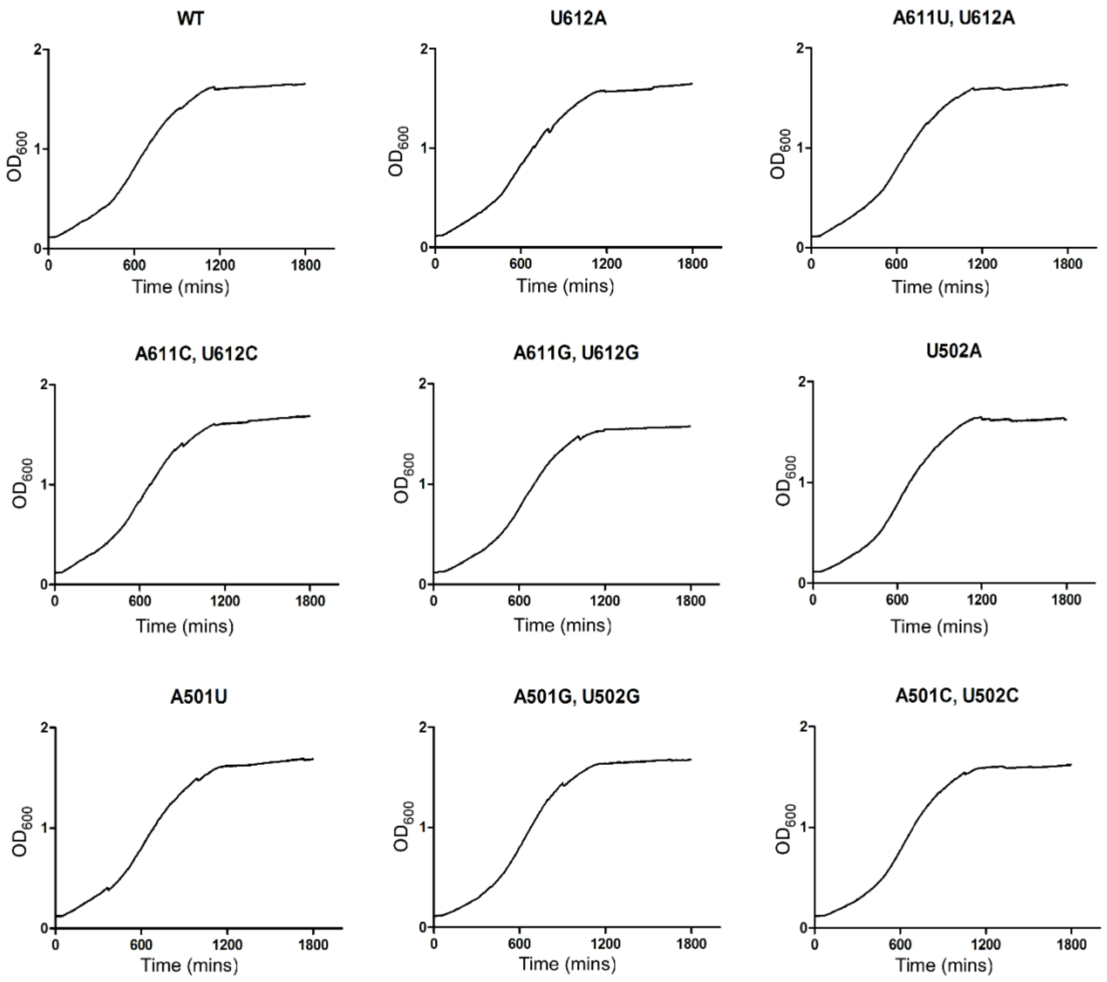

B

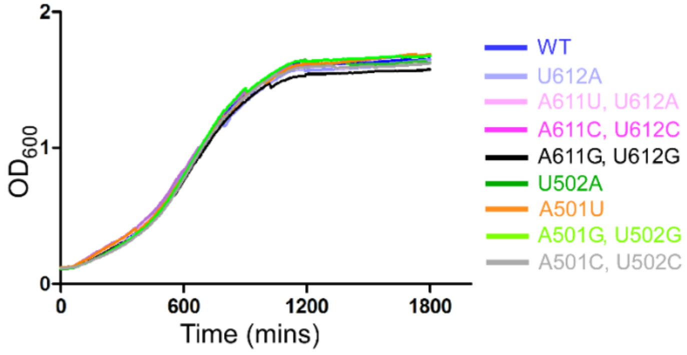

C

pDP333	Doubling time
WT	229 mins
U612A	226 mins
A611U, U612A	232 mins
A611C, U612C	228 mins
A611G, U612G	222 mins
U502A	212 mins
A501U	238 mins
A501G, U502G	206 mins
A501C, U502C	211 mins

Figure S3. (A-B). Representative growth curves of BY4741 cells expressing indicated mutants; **(C)** Doubling times based on the curves shown in A. Growth assays were performed in triplicates.

Supplemental Figure 4

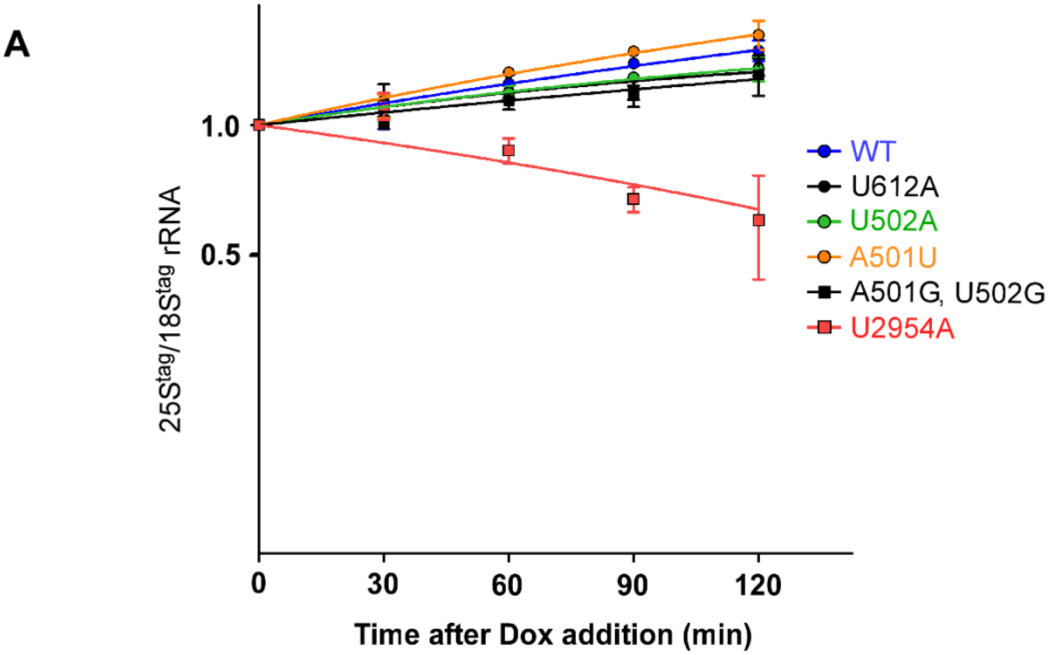

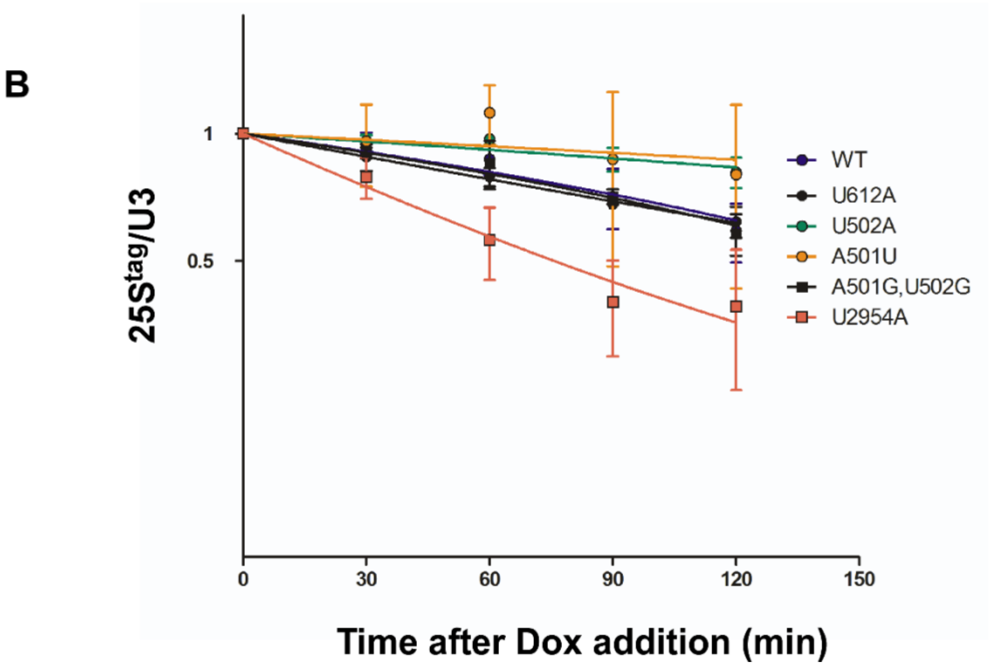

Figure S4. Additional assays for the comparative stability of the ES7L mutant rRNAs and the 25S-NRD substrate U2954. (A) 25S^{tag}/18S^{tag} rRNA ratios in each strain after Dox shut-down of the rRNA expression from pDP333. **(B)** 25S^{tag}/U3 snoRNA ratios. In both

assays, Dox was added to yeast cultures expressing the indicated $25S^{tag}$ rRNA mutants, and aliquots were collected at the indicated time points (0, 30, 60, 90, 120 min). RNA was extracted and analyzed by Northern hybridizations with the FL127, FL128 and U3 probes (Supplemental Table S1). Hybridization signals were quantified by phosphorimaging analysis and RNA ratios calculated for the values in the same lane. All ratios were normalized to t=0 min, the error bars show S.D., the Y axis is on the log scale. The small continuous increase in the $25S^{tag}/18S^{tag}$ rRNA ratio after Dox addition is intrinsic to the pDP333 expression system (see Figure S2). The values shown in this graph were used to calculate mutant $25S^{tag}$ rRNA levels relative to wild-type $25S^{tag}$ rRNA in Fig. 3. U3 snoRNA continues to be synthesized in the yeast growing cultures in the presence of Dox, accounting for the basal decline in the $25S^{tag}$/U3 snoRNA ratio over time for the wild-type pDP333 construct.

Supplemental Figure 5

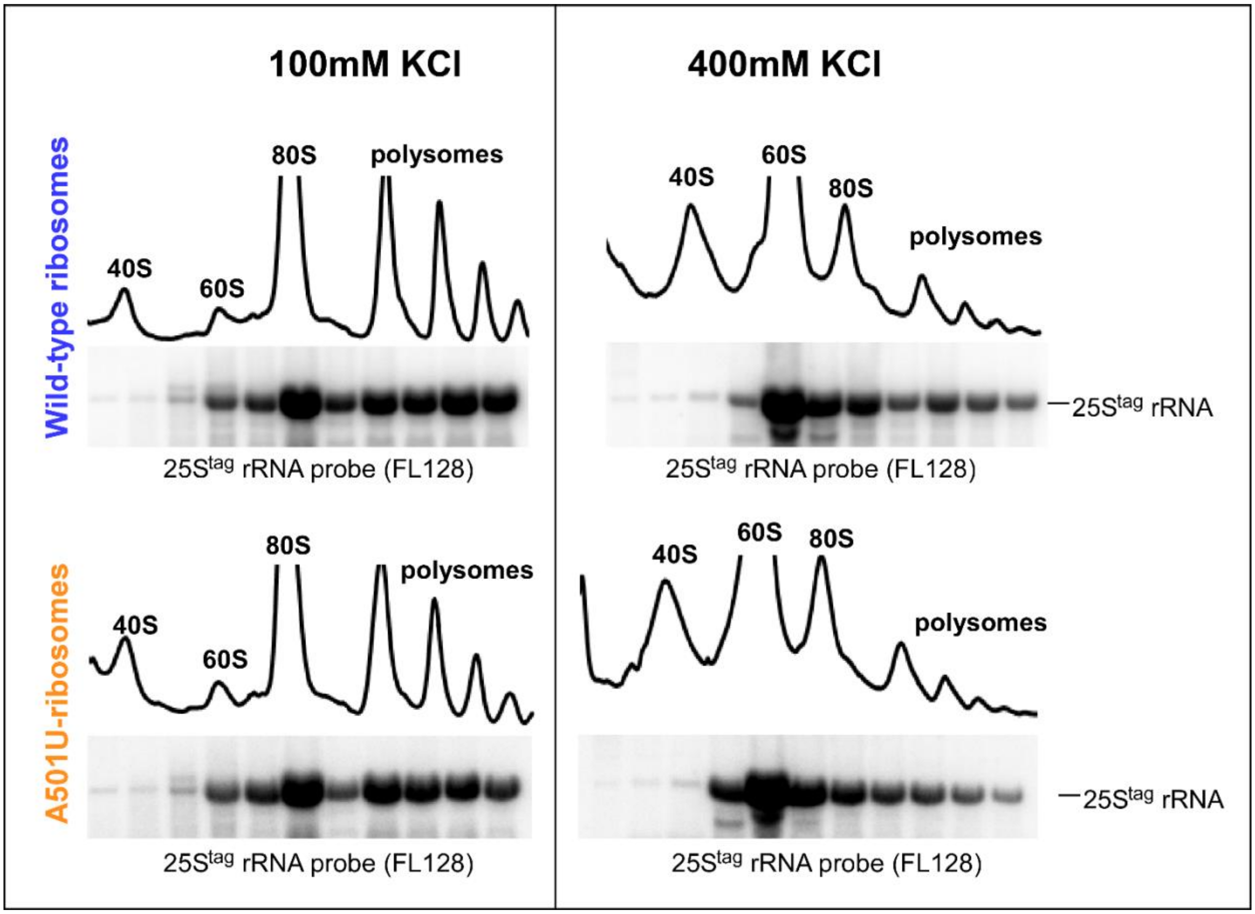

Figure S5. A501U-mutant and wild-type ribosomes behave in a similar fashion during high-salt gradient centrifugation. Sucrose gradient centrifugation analysis of polysomes from BY4741 cells expressing wild-type 25S^{tag} rRNA (top) or 25S^{tag}-A501U (bottom) rRNA, using low-salt (left) and high-salt (right) conditions. Cells were grown in the absence of Dox overnight and lysed in low-salt (100 mM KCl) or high-salt (400 mM KCl) buffer S (see Methods). Lysates were centrifuged through 15%-45% sucrose gradients. Gradients were fractionated with the continuous measurement of absorbance at 254 nm to visualize ribosomal peaks. Total RNA was extracted from individual fractions and analyzed by northern hybridizations with the radioactively labeled 25S^{tag} rRNA probe FL128.

Supplemental Figure 6

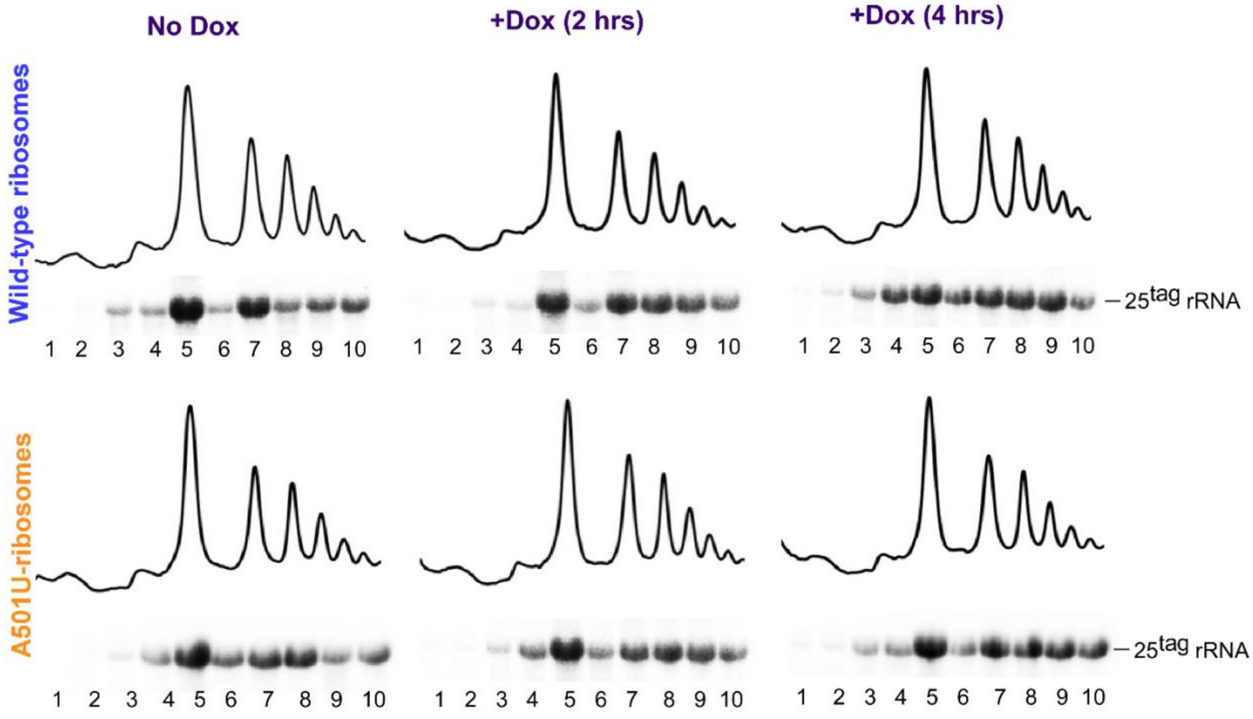

Figure S6. Both wild-type and A501U-ribosomes are present on polysomes after transcriptional shut-down. BY4741 transformed with pDP333 containing wild-type or mutant 25S^{tag} rRNAs were grown in the absence of Dox to induce tagged rRNA production. Dox was added to one half of the culture (+Dox) for 2 h or 4h, while another half remained Dox-free (no Dox). Cellular lysates prepared in buffer F (see Methods) were centrifuged through a sucrose gradient, and RNA extracted from individual fractions was analyzed by Northern hybridizations with the FL128 probe. Absorbance at 254 nm was used to visualize ribosomal peaks during gradient fractionation.

Supplemental Figure 7

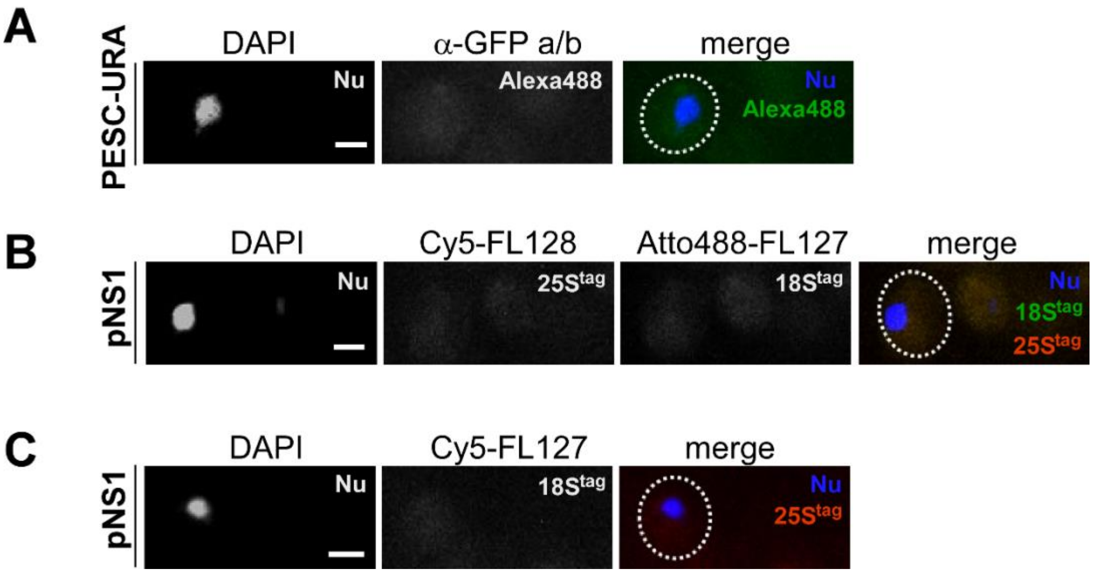

Figure S7. Cells transformed with an empty vector show no detectable nonspecific signal during protein immunostaining and FISH analyses. (A) BY4741 cells were transformed with PESC-URA, grown in synthetic dextrose medium in the absence of Dox, and shifted to galactose medium for 5 h. Immunostaining was done with anti-GFP primary and Alexa 488-conjugated secondary antibodies. DAPI was used to visualize the nucleus (Nu). Representative microscopy fields are shown. **(B and C)** BY4741 cells were transformed with pNS1 (*LEU2)*, grown in the absence of Dox and subjected to RNA-FISH using Cy5-FL128 and Atto 488-FL127 probes for the $25S^{tag}$ and $18S^{tag}$ rRNAs, respectively **(B),** or with the Cy5-FL127 probe for the $18S^{tag}$ rRNA **(C)**. Scale bar, 2μm.

Supplemental Figure 8

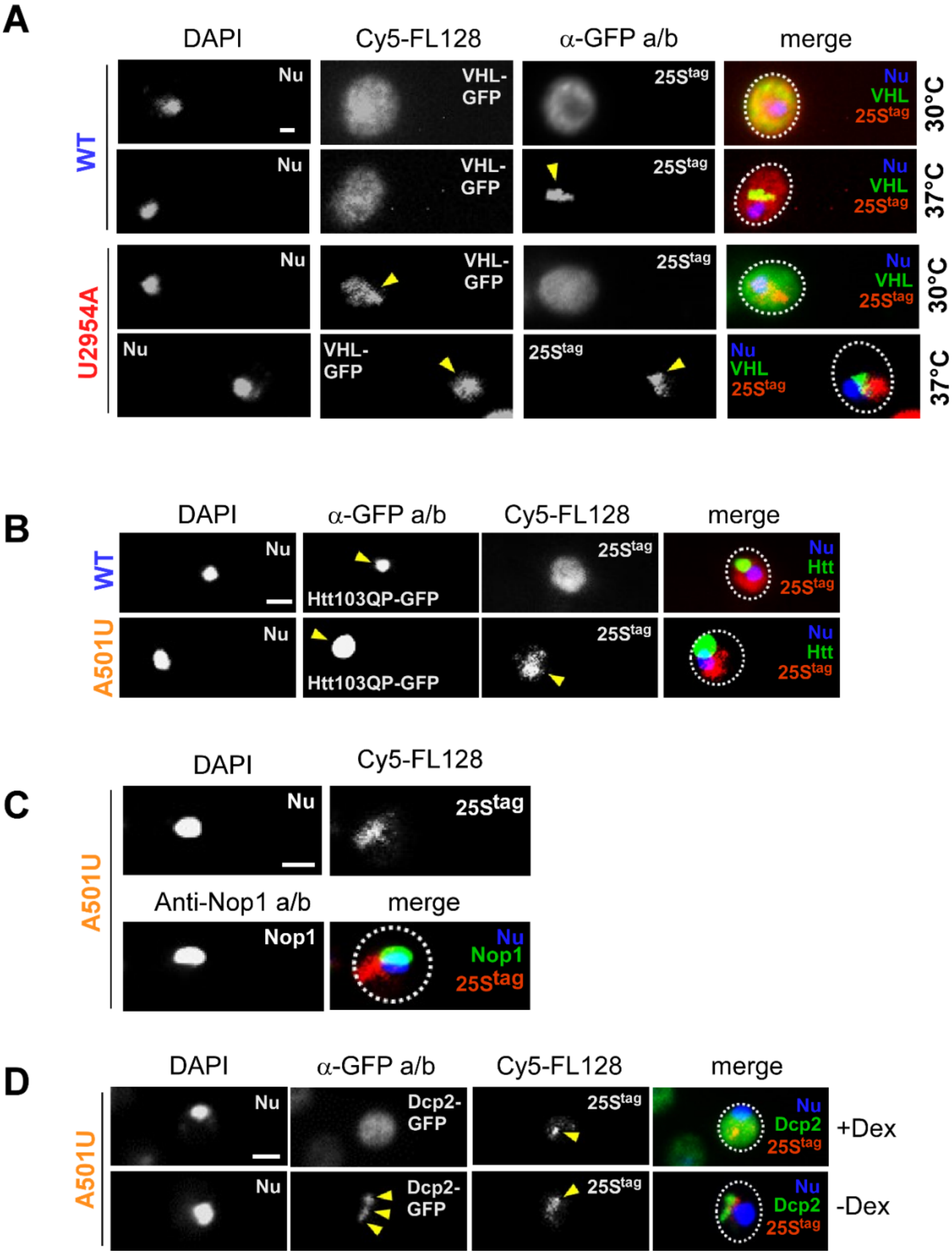

Figure S8. Ribosomes harboring the U2954A mutation do not colocalize with VHL. (A) BY4741 cells transformed with pDP333 carrying wild-type 25S^{tag} or 25S^{tag}-U2954A were cotransformed with PESC-URA-GFP-VHL. Overnight cultures grown in dextrose-containing SC-leu-ura- medium without Dox were shifted to galactose-containing medium for 4.5 h at 30°C, then shifted to 37°C or remained at 30°C for 30 min. VHL-GFP was visualized by immunostaining with anti-GFP and Alexa 488-conjugated antibodies, while 25S^{tag} rRNAs were detected by RNA-FISH using probe Cy5-FL128. DAPI was used to stain the nucleus (Nu). Representative microscopy fields are shown. Scale bar, 2 μm. **(B-D) Ribosomes harboring the A501U mutation do not colocalize with Htt103QP, Nop1 or Dcp2. (B)** BY4741 cells transformed with pDP333 carrying wild-type 25S^{tag} or 25S^{tag}-A501U were cotransformed with pYes-Htt103QP-GFP. Overnight cultures grown in dextrose-containing SD-leu-ura- medium without Dox were shifted to galactose-containing medium for 5 h. The distribution of 25S^{tag} rRNA was visualized by FISH using probe Cy5-FL128, followed by immunostaining with anti-GFP and Alexa 488-conjugated antibodies to detect Htt103QP-GFP. Yellow arrows point to A501U-ribosome-enriched foci and Htt103QP aggregates. DAPI was used to visualize the nucleus (Nu). Representative microscopy fields are shown. Scale bar, 2μm. **(C)** BY4741 cells expressing indicated 25S^{tag} rRNAs were analyzed by RNA-FISH with Cy5-FL128 probe, followed by immunostaining with anti-Nop1 and Alexa 488-conjugated antibodies. Scale bar, 2 μm. **(D)** BY4741 cells were transformed with pDP333 carrying 25S^{tag}-A501U and pRS313-DCP2-GFP. Transformants were grown overnight in SD-leu-his- in the absence of Dox, and one-half of the culture was incubated in medium lacking dextrose for 30 min. FISH and immunostaining were done as in (A). Yellow arrows point to A501U-ribosome-enriched foci and P-bodies. DAPI was used to visualize the nucleus (Nu). Representative microscopy fields are shown. Scale bar, 2μm.

SUPPLEMENTAL METHODS

Sucrose gradient centrifugation analysis.

For sucrose gradient centrifugation analysis, cells were pelleted by centrifugation, washed with water and lysed by glass bead shearing. Before harvesting, cell cultures were treated with 100 μg/ml CHX for 5 min. Cells were lysed in two alternative buffers as indicated in the figure legends. We used buffer F (10 mM Tris-HCl, pH 7.4, 100 mM NaCl, 3 mM $MgCl_2$, 100 μg/ml CHX, 200 μg/ml heparin) (Mansour and Pestov 2013b) and buffer S (10 mM Tris-HCl, pH 7.5, 100 or 400 mM KCl, 5 mM $MgCl_2$, 100 μg/ml CHX, 6 mM β-mercaptoethanol) (8). Lysates were clarified by centrifugation, and aliquots corresponding to 50 A_{260} units were loaded onto 15%–45% (w/v) sucrose gradients (11 ml). For centrifugation of lysates obtained with buffer F, we used sucrose solutions prepared in 10 mM Tris-HCl, pH 7.4, 70 mM NH_4Cl, 4 mM $MgCl_2$ (7); for centrifugation of lysates obtained with buffer S (containing 100 or 400 mM KCl) we used sucrose solutions prepared in the same buffer S. Gradients were centrifuged at 188,000xg at 4 °C for 4 h 15 min (Beckman SW41Ti rotor, 36,000 rpm), fractionated using a Beckman fraction recovery system connected to an EM-1 UV monitor (Bio-Rad), and A_{260} traces were digitally recorded using Windaq data acquisition software (DATAQ Instruments). Sucrose fractions were treated with 100 μg/ml proteinase K (Roche Life Science) in the presence of 16.5 μM EDTA and 1% SDS for 30 min at 42°C, followed by phenol/chloroform extraction and isopropanol precipitation. RNA pellets were resuspended in the FAE (Formamide, EDTA) solution (9) and analyzed by Northern hybridizations.

RNA extraction, northern blotting and signal quantification.

Total RNA was isolated from cells using one-step FAE extraction as described previously (9). RNA was resolved on 1.2% agarose gels containing 1.3% formaldehyde (10), transferred to nylon membranes (Hybond N+; GE Healthcare Life Sciences) and hybridized with ^{32}P-labeled oligonucleotide probes as described in (11). Sequences of all probes used in this study are listed in Table S1. Hybridizations were analyzed using an Amersham Typhoon 5 Biomolecular Imager

(GE Healthcare Life Sciences) and ImageQuant software. Signal quantification was done as described in (12). In brief, the volume of the hybridization signal corresponding to areas of interest was converted to phosphorimaging units using ImageQuant, and the average image background was subtracted.

Quantification of the relative content of the endogenous and tagged rRNA.

Individual colonies of BY4741 cells transformed with pDP333 constructs were grown in SC-leu at 30°C overnight, diluted in fresh SC-leu to an OD_{600}=0.3 and grown until the OD_{600}=0.7-0.8. Total RNA was extracted with FAE and additionally purified with RNazol RT as described (13). Aliquots containing 6.4 µg RNA were treated in 30-µl reactions with 2 units of RNase-free DNase I and 20 units of RiboLock (both from Thermo Scientific) for 30 min at 37°C. The reactions were stopped with 4 mM EDTA at 65°C for 5 min, cleaned up with Sera-Mag carboxylate-modified SpeedBeads (GE LifeSciences), RNA was eluted with 1 mM sodium citrate and its concentration was determined using a Qubit fluorometer (Thermo Fisher). cDNA was synthesized using 15 ng DNA-free RNA, 50 pmoles random nanomers and 100 units of the RevertAid reverse transcriptase (Thermo Scientific) in 10-µl reactions at 42°C for 45 min following the manufacturer's protocol. The reactions were heated to 70°C for 10 min, diluted 1:3 with water and 1 µl was used for each of the following 15-µl PCR reactions. As a control for DNase treatment, we performed a set of separate reactions without reverse transcriptase; all these reactions showed no detectable amplification in our PCR.

We designed PCR primer combinations to distinguish between endogenous (untagged) and plasmid-derived (tagged) rRNA. For endogenous rRNAs, we used primer pairs 25S-A, 25S-B and 18S-C, 18S-D, whereas for the tagged rRNAs, we used 25S-A, FL128-B and FL127-C, 18S-D (Supplemental table S1). All primer pairs were validated for the absence of cross-amplification of noncognate rRNAs for at least 25 PCR cycles. qPCR was performed with the PowerUp SYBR Green Master Mix (Thermo Scientific) using the annealing temperature of 54°C and a 30 s extension at 72°C, otherwise following the manufacture's standard cycling protocol on a

Mastercycler ep realplex (Eppendorf). Serial 1:5 dilutions of the Not I-digested plasmids pJD694 and pDP333 were used to obtain calibration curves for the nontagged and tagged rRNAs, respectively, built with linear regression analysis tools in GraphPad Prism 8.3. The values interpolated from the standard curves were used to calculate absolute molar ratios of the rRNAs in the cell-derived cDNA samples. Each cDNAs was analyzed in triplicate qPCR reactions.

RT-qPCR of mRNA in accordance with MIQE guidelines (14).

Experimental design: **a)** the experimental group consisted of samples expressing 25S^{tag}-A501U rRNA from the pDP333 plasmid, while the control group samples were those that expressed wild-type 25S^{tag} rRNA from the pDP333 plasmid; **b)** both the experimental and the control groups were derived from yeast cells expressing wild-type 25S^{tag} rRNA. The experimental group was treated with 50 μM BZ for 1 h at 30 °C, while in the control group drug treatment was omitted. Each sample represented an aliquot of yeast culture with an approximate volume of 50 μl. Prior to RNA extractions, cells were washed twice with water to remove medium and drugs.

Total cellular RNA was extracted using the FAE method (13). The concentration of the RNA was estimated spectrophotometrically; a total of 10 μg of RNA was treated with 2 units of DNase (Thermo Scientific, cat# EN0521) for 15 min at 37°C, purified by phenol/chloroform extraction, precipitated with isopropanol, resuspended in 15 μl of water and the concentration was re-measured (A_{260}/A_{280} ~ 2.1). To assess RNA quality, 1 μg RNA was separated on 1.2% agarose gel under denaturing conditions (15), stained with SYBR Gold (Thermo Scientific, cat#S11494), visualized using Amersham Typhoon 5 Biomolecular Imager (GE Healthcare Life Sciences) and evaluated with ImageQuant software. The reverse transcription reaction was done as follows: 1.5 μg of RNA was annealed with 0.4 μM oligo(dT)$_{18}$ primer (Thermo Scientific, cat# SO132) at 65 °C for 5 min in a total volume of 15 μl, transferred to 42 °C, and 10 μl of the master mix was immediatly added into the reaction. In each reaction, the master mix contained 200 units of the ReverseAid enzyme (Thermo Scientific, cat#EP0441), 1 mM dNTP mix (Thermo Scientific, cat#R0191), 40 units RiboLock RNase inhibitor (Thermo Scientific, cat# EO0384). As a DNA-

contamination control, one of the reactions for each set of primers had no ReverseAid enzyme. Reverse transcription reactions were incubated for 1.5 h at 42 °C, diluted with water up to 125 µl and the generated cDNA was either stored at -80 °C, or used immediately as a template in a real-time qPCR reaction on a Mastercycler ep realplex (Eppendorf). The PCR reaction volume was 15 µl, and contained 5 µl of cDNA, 0.4 µM forward (F), 0.4 µM of reverse (R) primers and 1x of RT-PCR NEW mix (Accuris™ qMax Green low Rox qPCR Mix, cat# PR2000-L). Primers were designed using Applied Biosystems software. The thermocycling parameters were as follows: the initial denaturation time was 10 min at 95 °C, followed by 40 cycles of: 95 °C for 15 sec (denaturation), 54 °C for 15 sec (annealing) and 72 °C for 30 sec (amplification). All samples were analyzed in triplicate, and *ACT1* was used to normalize the mRNA expression levels. At least three biological replicas were done for each experiment.

Table S3. Primers used in RT-qPCR analysis to detect proteotoxic stress markers.

Gene symbol	Sequence accession number	Primer sequences	Length of an amplicon
HSP12	Primary (citable) accession number: **P22943** Secondary accession number(s): D6VTL5, Q8X145	F 5′-TTTGGCAGACCAAGCTAGAGATT -3′<br>R 5′-CGGCATCGTTCAACTTGGA -3′	59 nt
HSP26	Primary (citable) accession number: **P15992** Secondary accession number(s): D6VQ71, Q6B1V5	F 5′-TGGGTGAAGGCGGCTTAA -3′<br>R 5′-GCGGGTGTGTTTGCTAACTGA -3′	57 nt
HSP42	Primary (citable) accession number: **Q12329** Secondary accession number(s): D6VSF3	F 5′-CTATTACTATAGTCCTGAAT -3′<br>R 5′- ATTCAGGACTATAGTAATAG-3′	60 nt
TMC1	Primary (citable) accession number: **Q08422** Secondary accession	F 5′-GCATGAGATCGAGGATAAAAGCA-3′<br>R 5′-CGGTCCCGGAATCTGATTT -3′	61 nt

	number(s): D6W2B7, O00017		
SSA4	Primary (citable) accession number: **P22202** Secondary accession number(s): D3DM10	F 5′-TGAGGCCGTCGCTTATGG -3′ R 5′-ACGACTGGTCACCCGTTAAGA -3′	59 nt
ACT1	Primary (citable) accession number: **P60010** Secondary accession number(s): D6VTJ1 Q9P3X7	F 5′-ATGGATTCTGAGGTTGCTGCTT -3′ R 5′-TGTCTTGGTCTACCGACGATAG -3′	119 nt

REFERENCES:

1. Rakauskaite,R. and Dinman,J.D. (2006) An arc of unpaired 'hinge bases' facilitates information exchange among functional centers of the ribosome. *Mol. Cell. Biol.*, **26**, 8992–9002.

2. LaRiviere,F.J., Cole,S.E., Ferullo,D.J. and Moore,M.J. (2006) A late-acting quality control process for mature eukaryotic rRNAs. *Mol. Cell*, **24**, 619–626.

3. Oakes,M., Aris,J.P., Brockenbrough,J.S., Wai,H., Vu,L. and Nomura,M. (1998) Mutational analysis of the structure and localization of the nucleolus in the yeast Saccharomyces cerevisiae. *J. Cell Biol.*, **143**, 23–34.

4. Meriin,A.B., Zhang,X., Alexandrov,I.M., Salnikova,A.B., Ter-Avanesian,M.D., Chernoff,Y.O. and Sherman,M.Y. (2007) Endocytosis machinery is involved in aggregation of proteins with expanded polyglutamine domains. *FASEB J. Off. Publ. Fed. Am. Soc. Exp. Biol.*, **21**, 1915–1925.

5. Segal,S.P., Dunckley,T. and Parker,R. (2006) Sbp1p affects translational repression and decapping in Saccharomyces cerevisiae. *Mol. Cell. Biol.*, **26**, 5120–5130.

6. Shcherbik,N. (2013) Golgi-mediated glycosylation determines residency of the T2 RNase Rny1p in Saccharomyces cerevisiae. *Traffic Cph. Den.*, **14**, 1209–1227.

7. Shcherbik,N., Chernova,T.A., Chernoff,Y.O. and Pestov,D.G. (2016) Distinct types of translation termination generate substrates for ribosome-associated quality control. *Nucleic Acids Res.*, **44**, 6840–6852.

8. van den Elzen,A.M.G., Schuller,A., Green,R. and Séraphin,B. (2014) Dom34-Hbs1 mediated dissociation of inactive 80S ribosomes promotes restart of translation after stress. *EMBO J.*, **33**, 265–276.

9. Shedlovskiy,D., Shcherbik,N. and Pestov,D.G. (2017) One-step hot formamide extraction of RNA from Saccharomyces cerevisiae. *RNA Biol.*, **14**, 1722–1726.

10. Mansour,F.H. and Pestov,D.G. (2013) Separation of long RNA by agarose-formaldehyde gel electrophoresis. *Anal. Biochem.*, **441**, 18–20.

11. Pestov,D.G., Lapik,Y.R. and Lau,L.F. (2008) Assays for ribosomal RNA processing and ribosome assembly. *Curr. Protoc. Cell Biol.*, **Chapter 22**, Unit 22.11.

12. Shedlovskiy,D., Zinskie,J.A., Gardner,E., Pestov,D.G. and Shcherbik,N. (2017) Endonucleolytic cleavage in the expansion segment 7 of 25S rRNA is an early marker of low-level oxidative stress in yeast. *J. Biol. Chem.*, **292**, 18469–18485.

13. Shedlovskiy,D., Shcherbik,N. and Pestov,D.G. (2017) One-step hot formamide extraction of RNA from Saccharomyces cerevisiae. *RNA Biol.*, **14**, 1722–1726.

14. Bustin,S.A., Benes,V., Garson,J.A., Hellemans,J., Huggett,J., Kubista,M., Mueller,R., Nolan,T., Pfaffl,M.W., Shipley,G.L., *et al.* (2009) The MIQE guidelines: minimum information for publication of quantitative real-time PCR experiments. *Clin. Chem.*, **55**, 611–622.

15. Mansour,F.H. and Pestov,D.G. (2013) Separation of long RNA by agarose-formaldehyde gel electrophoresis. *Anal. Biochem.*, **441**, 18–20.
